# Vibration analysis and pull-in instability behavior in a multiwalled piezoelectric nanosensor with fluid flow conveyance

**DOI:** 10.3762/bjnano.11.92

**Published:** 2020-07-21

**Authors:** Sayyid H Hashemi Kachapi

**Affiliations:** 1 Department of Mechanical Engineering, Babol Noshirvani University of Technology, P.O. Box 484, Shariati Street, Babol, Mazandaran 47148-71167, Iran

**Keywords:** electrostatic excitation, piezoelectric nanosensor, pull-in voltage, stability analysis, surface/interface effect, van der Waals force, viscous fluid velocity

## Abstract

In this work, surface/interface effects for pull-in voltage and viscous fluid velocity effects on the dimensionless natural frequency of fluid-conveying multiwalled piezoelectric nanosensors (FC-MWPENSs) based on cylindrical nanoshells is investigated using the Gurtin–Murdoch surface/interface theory. The nanosensor is embedded in a viscoelastic foundation and subjected to nonlinear van der Waals and electrostatic forces. Hamilton’s principle is used to derive the governing and boundary conditions and is also the assumed mode method used for changing the partial differential equations into ordinary differential equations. The influences of the surface/interface effect, such as Lame’s constants, residual stress, piezoelectric constants and mass density, are considered for analysis of the dimensionless natural frequency with respect to the viscous fluid velocity and pull-in voltage of the FC-MWPENSs.

## Introduction

Nanomechanical sensors and resonators, especially when combined with piezoelectric materials, are widely used in modern engineering, which encompasses numerous, diverse fields of science and technology, pharmaceutical, agricultural, environmental, advanced materials, chemical science, physics, electronics, information technology, biomedical and medical fields [1–10]. Due to this extended use of nanosensors, especially piezoelectric nanosensors in vibration devices, mathematical models and the study of vibration behavior are essential. Additionally, it is important that the size-dependent parameters for the dynamics analysis and mathematical modeling of these nanostructures be contained in the theoretical models. For this reason, surface/interface elasticity, which was addressed by Gurtin and Murdoch, is taken into consideration [11]. Also, multiwalled nanoshell (MWNS) materials are structurally built by multiconcentric single-walled nanoshell (SWNS) materials, and the mechanical properties of MWNA materials have been found to be superior to the mechanical properties of SWNSs. As a result, MWNS materials are preferred in many applications such as nanoresonators.

Many studies have been carried out on the vibration and stability analysis of nanostructures with some reviews given as follows. Strozzi and Pellicano investigated the vibration analysis of triple-walled carbon nanotubes (TWNTs) subjected to the interlayer van der Waals (vdW) force in the framework of the Sanders–Koiter shell theory [12]. Also, based on nonlocal cylindrical shell theory, Ghorbanpour Arani et al. studied nonlinear vibration and instability of double-walled boron nitride nanotubes [13]. Malihi et al. investigated the effect of nonzero initial conditions, the nonlinear coefficient of squeeze film air damping, and the van der Waals effect on the stability of torsional nanomirrors for the obtained dynamic pull-in instability voltage using the size effect [14]. Fakhrabadi et al. utilized the modified couple stress theory to investigate the effects of various fluid parameters on the pull-in voltage of carbon nanotubes conveying viscous fluid [15]. Also, the vibration analysis of viscoelastic double-walled carbon nanotubes (DWCNTs) combined with ZnO layers and subjected to magnetic and electric fields were studied by Fereidoon et al. [16]. Recently, Hashemi Kachapi et al. presented a Gurtin–Murdoch surface/interface theory to investigate linear and nonlinear vibration analysis of piezoelectric nanostructures [17–20]. Free vibration of nanometer-sized piezoelectric double-shell structures and nonlinear buckling and postbuckling behavior of functionally graded piezoelectric cylindrical nanoshells were studied by Fang et al. using the surface energy effect [21–22]. Also, Zhu et al. utilized the surface energy effect to investigate a new approach for smart control of nonlinear free vibration of piezoelectric doubly curved nanoshells and orthotropic piezoelectric cylindrical nanoshells [23–24]. Wang utilized surface strain gradient elasticity to study a meticulous solution to the anti-plane shear problem of a circular elastic inhomogeneity [25]. Nami et al. utilized nonlocal elasticity theory and trigonometric shear deformation theory to investigate the static analysis of rectangular nanoplates [26]. The Gurtin–Murdoch surface theory is presented by Sigaeva et al. to study the universal model describing plane strain bending of a multilayered sector of a cylindrical tube [27]. Karimipour et al. presented a modified strain gradient theory (MSGT) and Gurtin–Murdoch surface elasticity to investigate the size-dependent nonlinear pull-in instability [28]. A new size-dependent nonlinear model for the analysis of the behavior of carbon nanotube resonators was introduced by Farokhi et al. based on modified couple stress theory [29]. Liu et al. utilized a new finite element method for modeling thin structures with surface effects by using layered shell elements [30].

To the best knowledge of the author, the surface/interface effect on pull-in voltage, viscous fluid velocity effects and dimensionless natural frequency (DNF) of multiwalled piezoelectric nanosensors conveying viscous fluid has not yet been studied. In the present study, the effect of surface/interface parameters such as Lame’s constants (λ^I,S^, µ^I,S^), residual stress 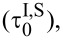 piezoelectric constants 
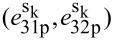
 and mass density (ρ^I,S^) are studied for analysis of dimensionless natural frequency with respect to viscous fluid velocity 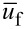 and pull-in voltage 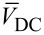 of fluid-conveying multiwalled piezoelectric nanosensors (FC-MWPENSs) subjected to direct electrostatic DC voltage with nonlinear excitation, nonlinear van der Waals force and viscoelastic foundation. As a guide to the reader, all notation and symbols are presented in Table 1.

**Table 1:** Notation and symbols used in this work.

Symbol	Description	Symbol	Description

*h*_Nn_	thickness of nanoshell	*h*_pn_	piezoelectric layer thickness
*L*	piezoelectric nanoshell length	*E*_pn_	Young modulus of piezoelectric layer
*R*_n_	the mid-surface radius	υ_pn_	Poisson’s ratio of piezoelectric layer
*x*	axial direction	ρ_p_	mass density of piezoelectric layer
θ	circumferential direction	e_31pn_, e_32pn_	piezoelectric constants
*z*	radius direction	η_33pn_	dielectric constant
*E*_Nn_	Young’s modulus of nanoshell	*s*_kn_	piezoelectric inner and outer surface
υ_Nn_	Poisson’s ratio of nanoshell	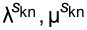	Lame’s constants of piezoelectric layer
ρ_Nn_	mass density of nanoshell	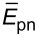	electric field
*I*_kn_	nanoshell inner and outer surface	*D*_zpn_	electric displacement
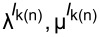	Lame’s constants of nanoshell	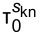	residual stress of piezoelectric layer
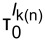	residual stress of nanoshell	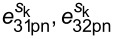	surface piezoelectric constants
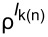	nanoshell interface mass density	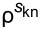	piezoelectric surface mass density
*C*_ijNn_CijNn	elastic constant of nanoshell	*C*_ijpn_	elastic constant of piezoelectric layer
σijNn	middle stress of nanoshell	σ_ijpn_	middle stress of piezoelectric layer
κ_(x,θ)_	curvature components	*V*_pn_	piezoelectric voltage
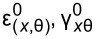	middle surface strains	π_n_	total strain energy
*u*	displacement of x direction	*T*_n_	total kinetic energy
*v*	displacement of θ direction	*I*_n_	mass moments of inertia
*w*	displacement of z direction	*C*_wn_	Damping coefficient
∇	Laplace operator	*K*_wn_	Winkler modulus
ω	natural frequency	*K*_pn_	Pasternak shear modulus
*M*	total mass matrix	*W*_n_	total work
*C*	total damping coefficient	*K*_n_	total stiffness matrix
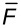	load by piezoelectric voltage	*b*_n_	nanosensor gap width
*V*_DnC_	direct electric voltage	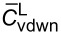	linear van der Waals coefficient
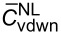	nonlinear van der Waals coefficient		

## Mathematical Formulation

**Figure 1:**
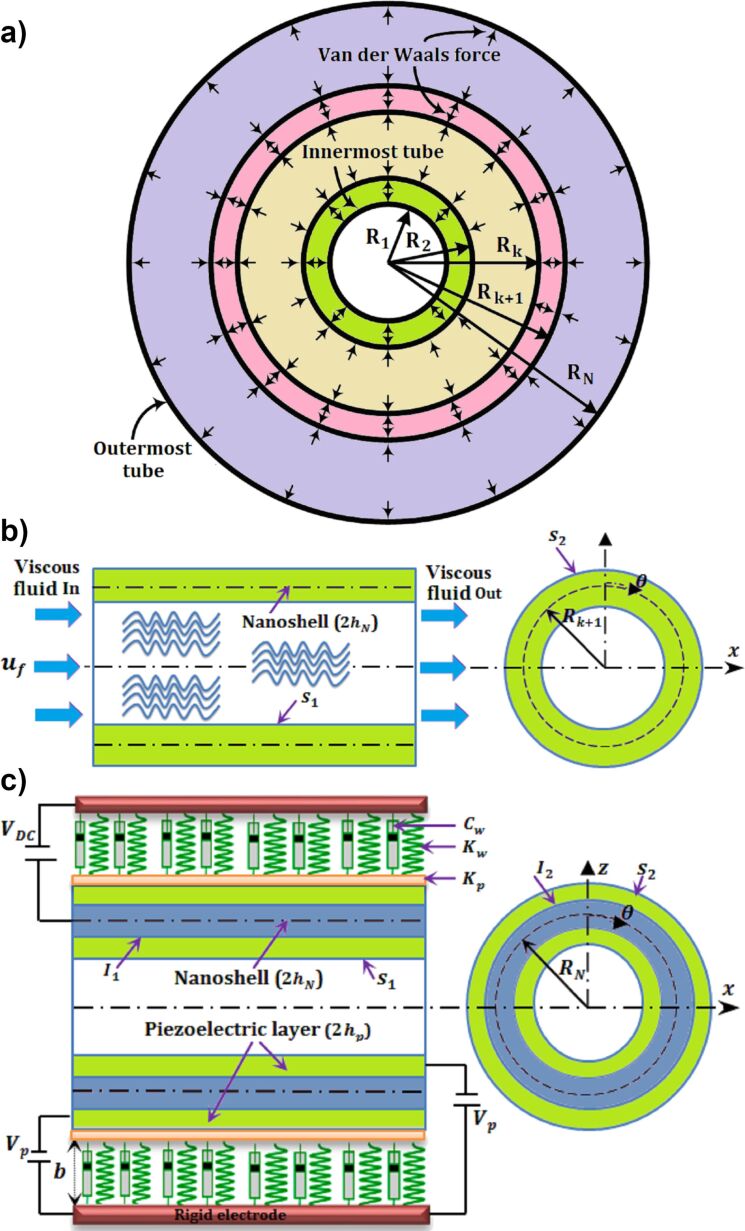
Fluid-conveying multiwalled piezoelectric nanosensor. (a) Illustration of van der Walls forces between two adjacent tubes of a multiple shell cross section of a multiwalled carbon nanotube (MWCNT). (b) Modeling of a 1…*k* + 1 tube of a MWCNT as a fluid-conveying nanosensor with surface model. (c) Modeling of the last tube of a MWCNT as a piezoelectric nanosensor with surface/interface model.

A schematic diagram of a multiwalled piezoelectric nanosensor with an embedded fluid-conveying inner layer, two piezoelectric layers, and a viscoelastic foundation medium in the outer layer is shown in Figure 1a–c. The geometrical parameters of the cylindrical shell are the length *L*, the mid-surface radius *R*_n_ with nanoshell thickness 2*h*_Nn_ and coated by two piezoelectric layers with total thickness 2*h*_pn_ for the outer wall, and also the mid-surface radius *R*_k+1_ and nanoshell thickness 2*h*_N(k+1)_ for the other inner wall layers. All of the physical and geometrical properties of the mentioned nanostructures for single-walled piezoelectric nanoresonators can be seen in work done by Hashemi Kachapi et al. in reference [18,20].

The governing equations and the solution procedure can be found in Supporting Information File 1.

## Results and Discussion

A verification study is investigated in work by Hashemi Kachapi et al. [18–20] with full details for single-walled (SW) and double-walled (DW) piezoelectric nanostructures. In this section, the effects of surface/interface parameters of FC-MWPENS, such as Lame’s constants (λ^I,S^, µ^I,S^), residual stress 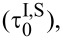 piezoelectric constants 
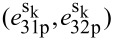
 and mass density (ρ^I,S^), are investigated for analysis of the dimensionless natural frequency with respect to viscous fluid velocity 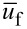 and pull-in voltage 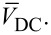 In order to simplify the presentation, CC, SS and CS represent the clamped edge, simply supported edge and clamped-simply supported edge, respectively. The material properties of the different layers of aluminum (Al) nanoshell and piezoelectric layers (PZT-4) are shown in Table 2 and Table 3, respectively [18,20].

**Table 2:** Surface and bulk properties of Al nanoshell.

*E*_N_ (GPa)	υ_N_	ρ_N_ (kg/m^3^)	λ^I^ (N/m)	µ^I^ (N/m)	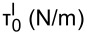	ρ^I^ (kg/m^2^)

70	0.33	2700	3.786	1.95	0.9108	5.46 × 10^−7^

**Table 3:** Surface and bulk properties of PZT-4 piezoelectric layers.

*C*_11p_ (GPa)	*C*_22p_ (GPa)	*C*_12p_ (GPa)	*C*_21p_ (GPa)	*C*_66p_ (GPa)	*E*_p_ (GPa)

139	139	77.8	77.8	30.5	95
υ_p_	ρ_p_ (kg/m^3^)	η_33p_ (10^−8^ F/m)	λ^S^ (N/m)	µ^S^ (N/m)	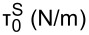
0.3	7500	8.91	4.488	2.774	0.6048
*e*_31p_ (C/m^2^)	*e*_32p_ (C/m^2^)	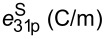	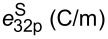	ρ^S^ (kg/m^2^)	
−5.2	−5.2	−3 × 10^−8^	−3 × 10^−8^	5.61 × 10^−6^	

The other bulk and surface geometrical parameters of FC-MWPENS are shown in Table 4 [18,20].

**Table 4:** The material and geometrical parameters of FC-MWPENS.

*R*_1_ (m)	*R*_2_ (m)	*R*_3_ (m)	*L*/*R*_1_	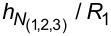

1 × 10^−9^	1.5 × 10^−9^	2 × 10^−9^	10	0.01
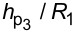	*b*_3_/*R*_3_	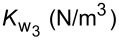	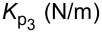	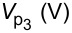
0.005	0.1	8.9995035 × 10^17^	2.071273	1 × 10^−3^
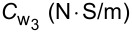	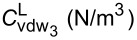	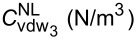	*V*_03_	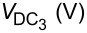
1 × 10^−3^	9.91866693 × 10^19^	2.201667 × 10^31^	1	5
µ_f_ (Pa·s)	ρ_f_ (kg/m^3^)	*u*_f_ (m/s)		
3 × 10^−3^	1060	50		

The value of the mid-surface radius for different PENS are presented as following: for single-walled PENS (SWPENS): *R* = *R*_out_; for double-walled PENS (DWPENS): *R*_1_ = *R*_in_, *R*_2_ = *R*_out_; and for triple-walled PENS (TWPENS): *R*_1_ = *R*_in_, *R*_2_ = *R*_mid_, *R*_3_ = *R*_out_.

### Surface/interface effects on dimensionless natural frequency with respect to viscous fluid velocity and pull-in voltage

In this section, the effect of surface/interface parameters, such as Lame’s constants (λ^I,S^, µ^I,S^), residual stress 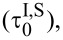 piezoelectric constants 
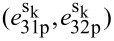
 and mass density (ρ^I,S^), of fluid-conveying multiwalled piezoelectric nanosensors are studied for analysis of the dimensionless natural frequency (DNF) with respect to viscous fluid velocity 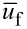 and pull-in voltage 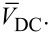 For this work, the material and geometrical parameters in Tables 2–4 are used. In all of the following results of the analysis of DNF on viscous fluid velocity 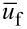 and pull-in voltage 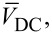 respectively, the values of 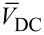 = 5 and 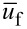 = 0.1 are used.

**Figure 2:**
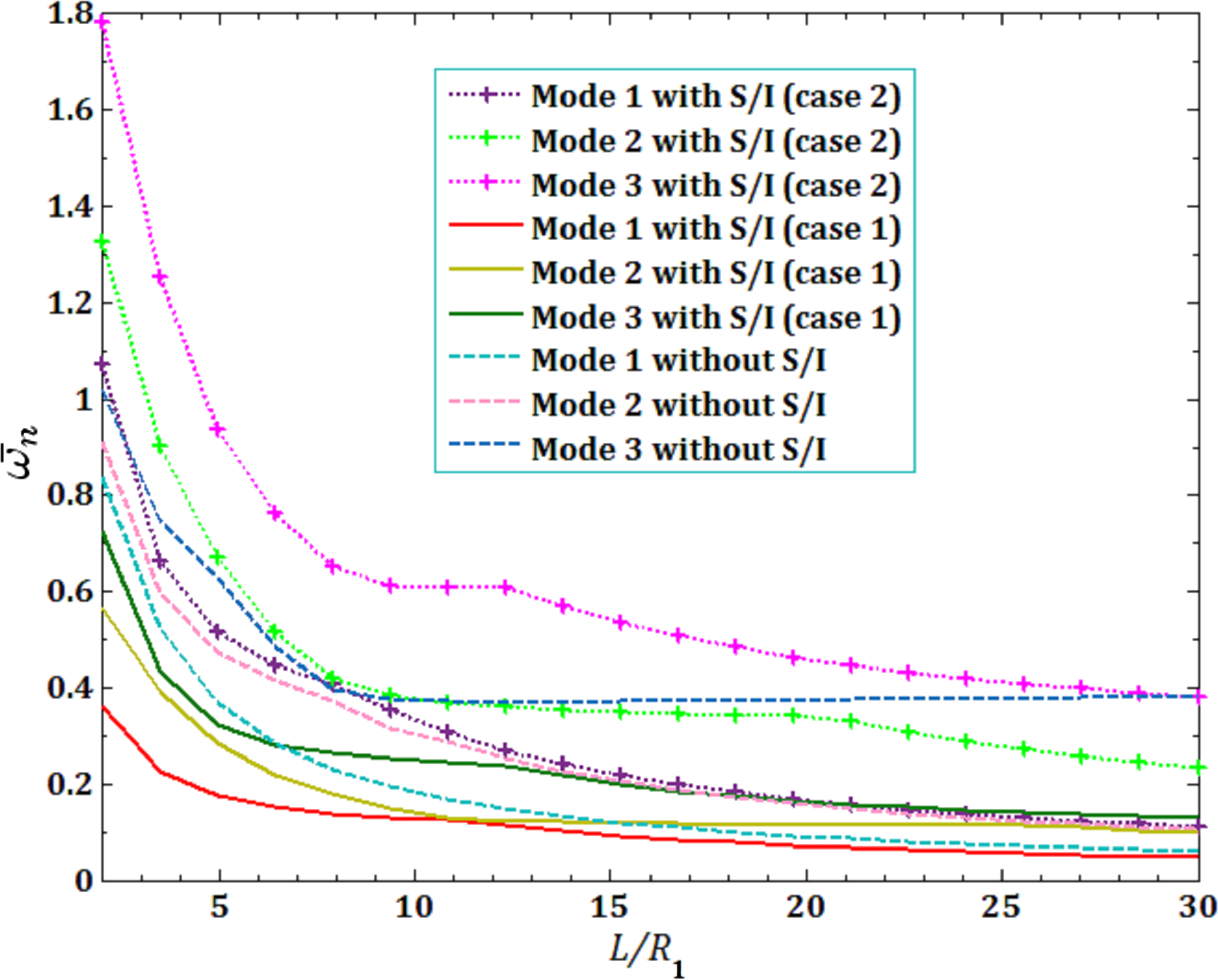
The surface/interface effects on DNF versus the *L*/*R*_1_ ratio for three vibrational modes.

First, the relationship between the DNF and the different MWPENR length-to-radius ratios *L*/*R*_1_ is shown in Figure 2 for three vibrational modes. These results are shown for two cases of surface density corresponding to Table 5 (due to fact the surface/interface density plays an important role in analysis of natural frequency and nonlinear frequency response).

**Table 5:** Two cases of surface density studied.

Case 1		Case 2	
ρ^I^ (kg/m^2^)	ρ^S^ (kg/m^2^)	ρ^I^ (kg/m^2^)	ρ^S^ (kg/m^2^)

5.46 × 10^−7^	5.61 × 10^−6^	5.46 × 10^−8^	5.61 × 10^−7^

It is observed that for all modes, the DNF decreases when the *L*/*R*_1_ ratio increases. Also, the DNF for mode 3 is higher than that for modes 1 and 2. It is clear from this figure that in the case of higher surface/interface density (case 1), the inertia of the shell is increased and its stiffness is reduced, which leads to a decreased DNF compared to the case without surface/interface effects. Also, with decreasing surface/interface density (case 2), the inertia of the system is increased, and with increasing stiffness, DNF increases compared to the case without surface/interface effects.

In all of the following results, the lower surface/interface density (case 2) is used in the analysis of DNF on viscous fluid velocity 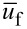 and pull-in voltage 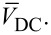


**Figure 3:**
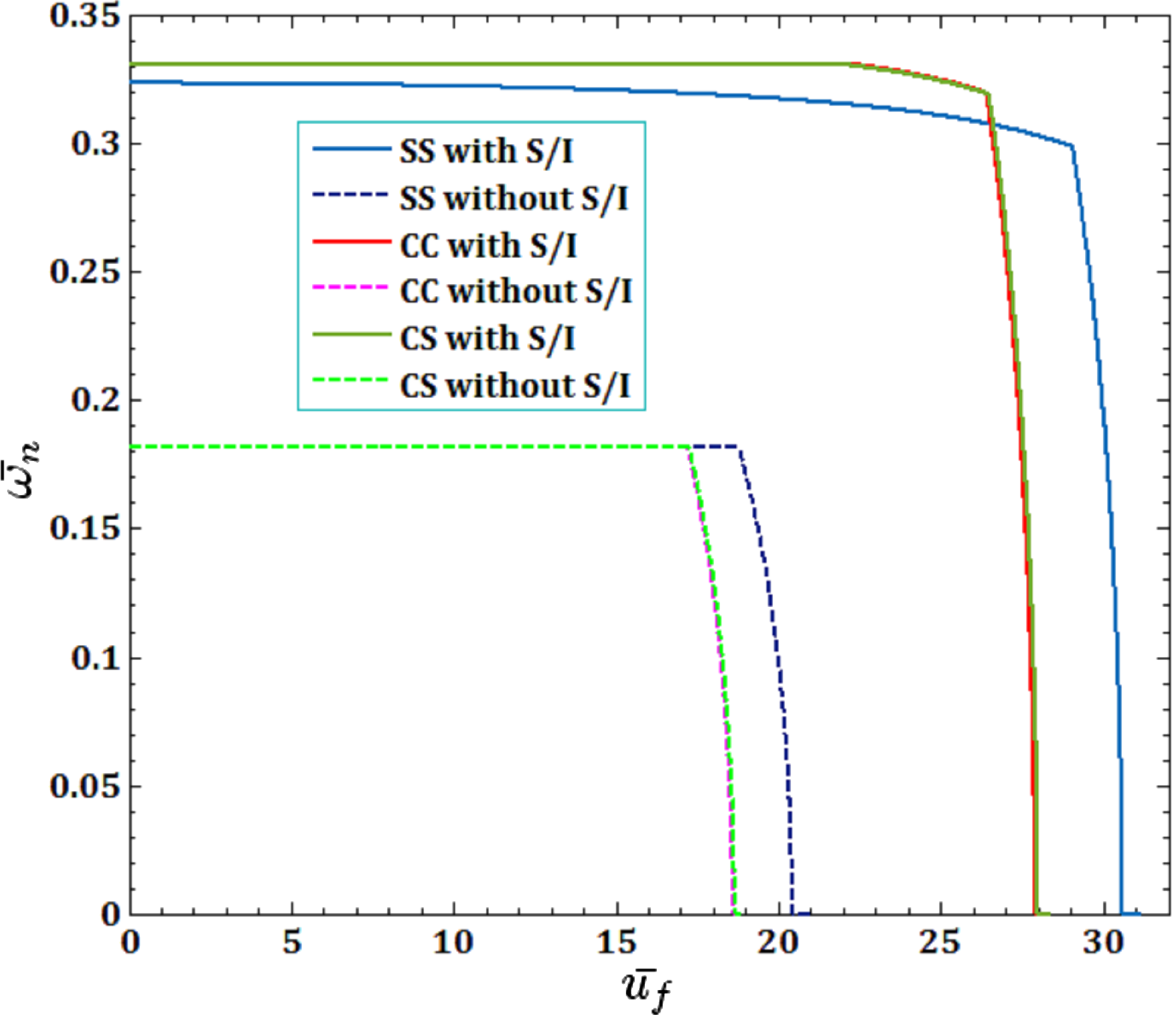
The effects of different boundary conditions for fluid velocity 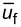 on DNF of FC-MWPENS.

**Figure 4:**
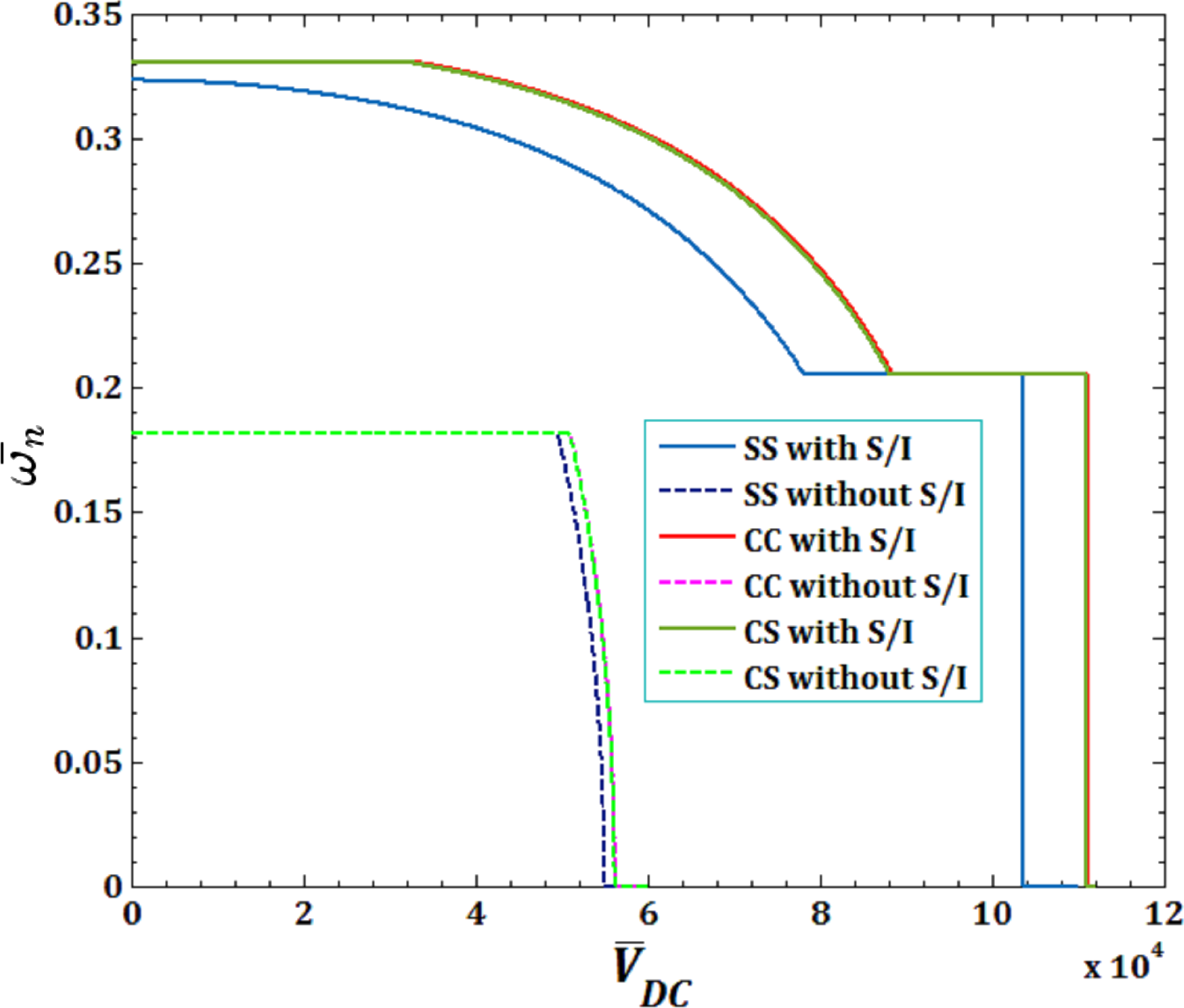
The effects of different boundary conditions for pull-in voltage on DNF of FC-MWPENS.

The effects of viscous fluid velocity 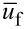 and direct pull-in DC voltage on the pull-in instability analysis on the DNF 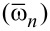 of FC-MWPENS are presented in Figure 3 and Figure 4 and for different boundary conditions. It can be seen that in all boundary conditions, the natural frequency decreases with increasing fluid velocity and voltage DC. Also, due to the system softening in the SS boundary condition (with and without surface/interface and low natural frequency in this case), FC-MWPENS is at a higher critical fluid velocity and lower pull-in voltage than other boundary conditions. After the SS boundary condition, the other boundary conditions CS and CC reach the zero due to being softer. For zero natural frequency, FC-MWPENS becomes unstable and this physically implies that the FC-MWPENS loses its stability due to the divergence via a pitchfork bifurcation.

**Figure 5:**
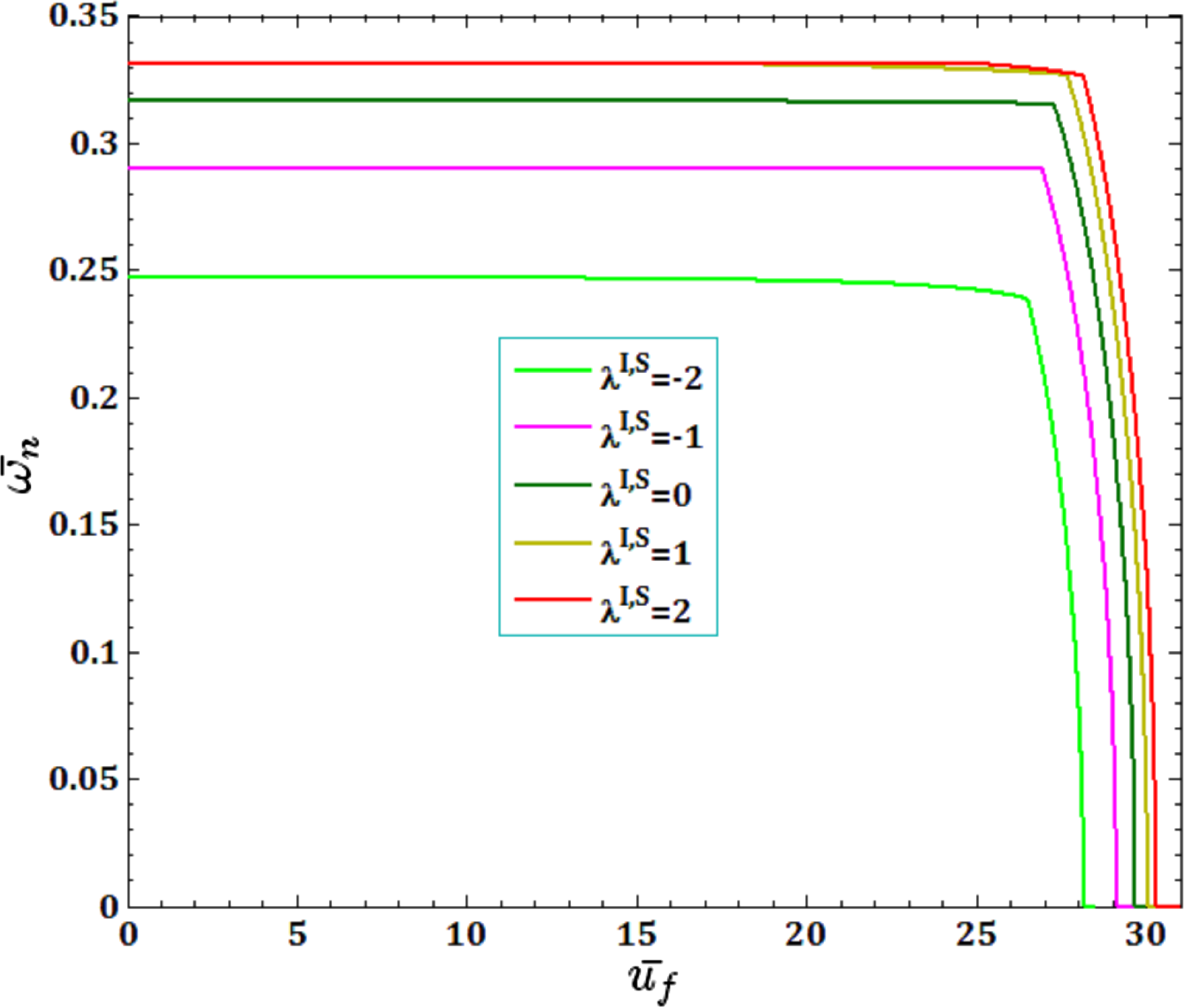
The effects of surface/interface Lame’s constants λ^I,S^ for fluid velocity 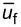 on DNF of SS FC-MWPENS.

**Figure 6:**
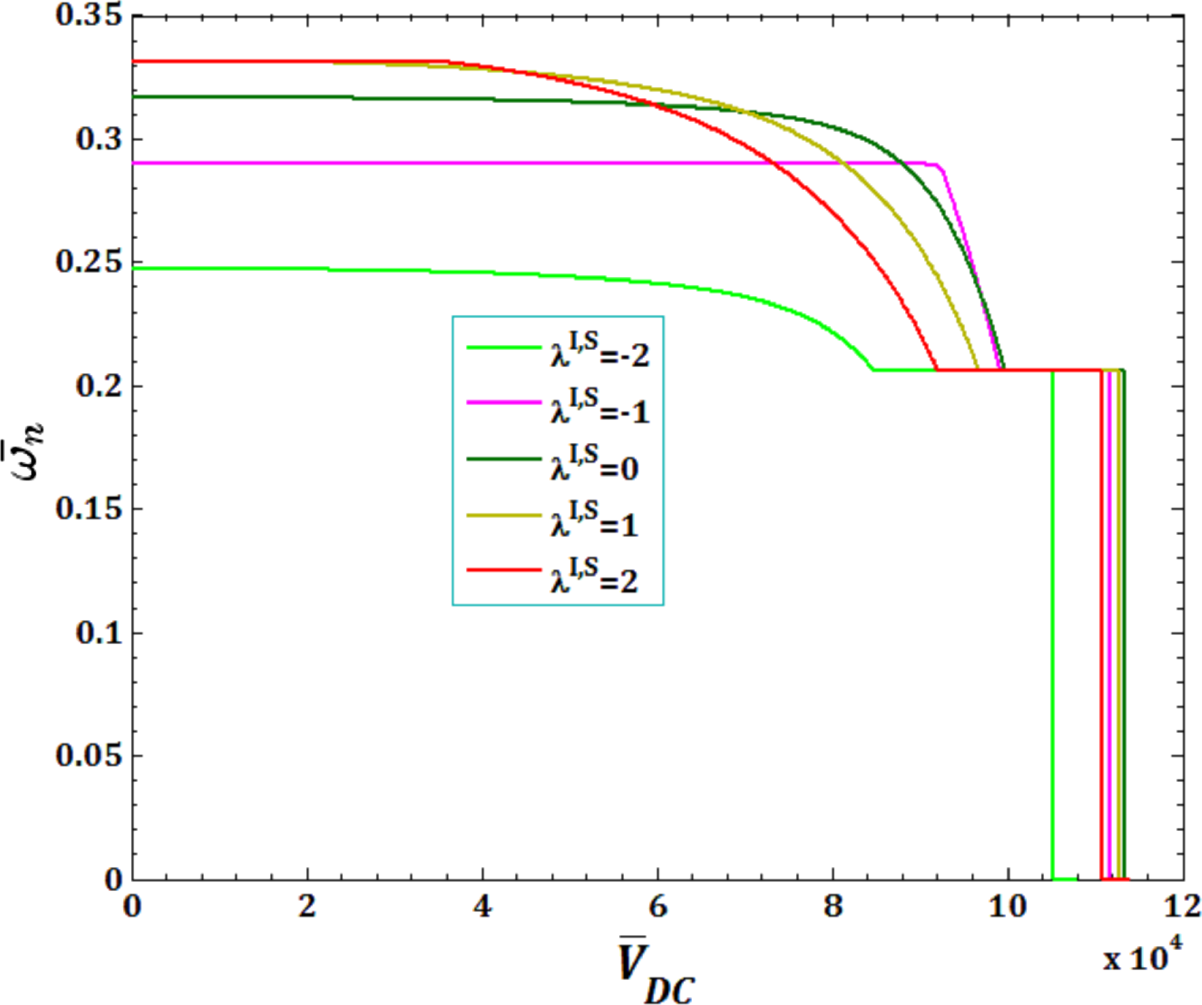
The effects of surface/interface Lame’s constants λ^I,S^ for pull-in voltage on DNF of SS FC-MWPENS.

Figure 5 and Figure 6 illustrate the effects of different surface and interface Lame’s constants, 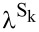 and 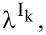 for viscous fluid velocity 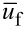 and pull-in instability analysis on DNF of FC-MWPENS. It is clear that the increasing surface/interface Lame’s constants λ^I,S^, due to increasing FC-MWPENS stiffness, DNF and critical fluid velocity increase and pull-in voltage in λ^I,S^ = 0 and λ^I,S^ = −2 has a maximum and a minimum value.

**Figure 7:**
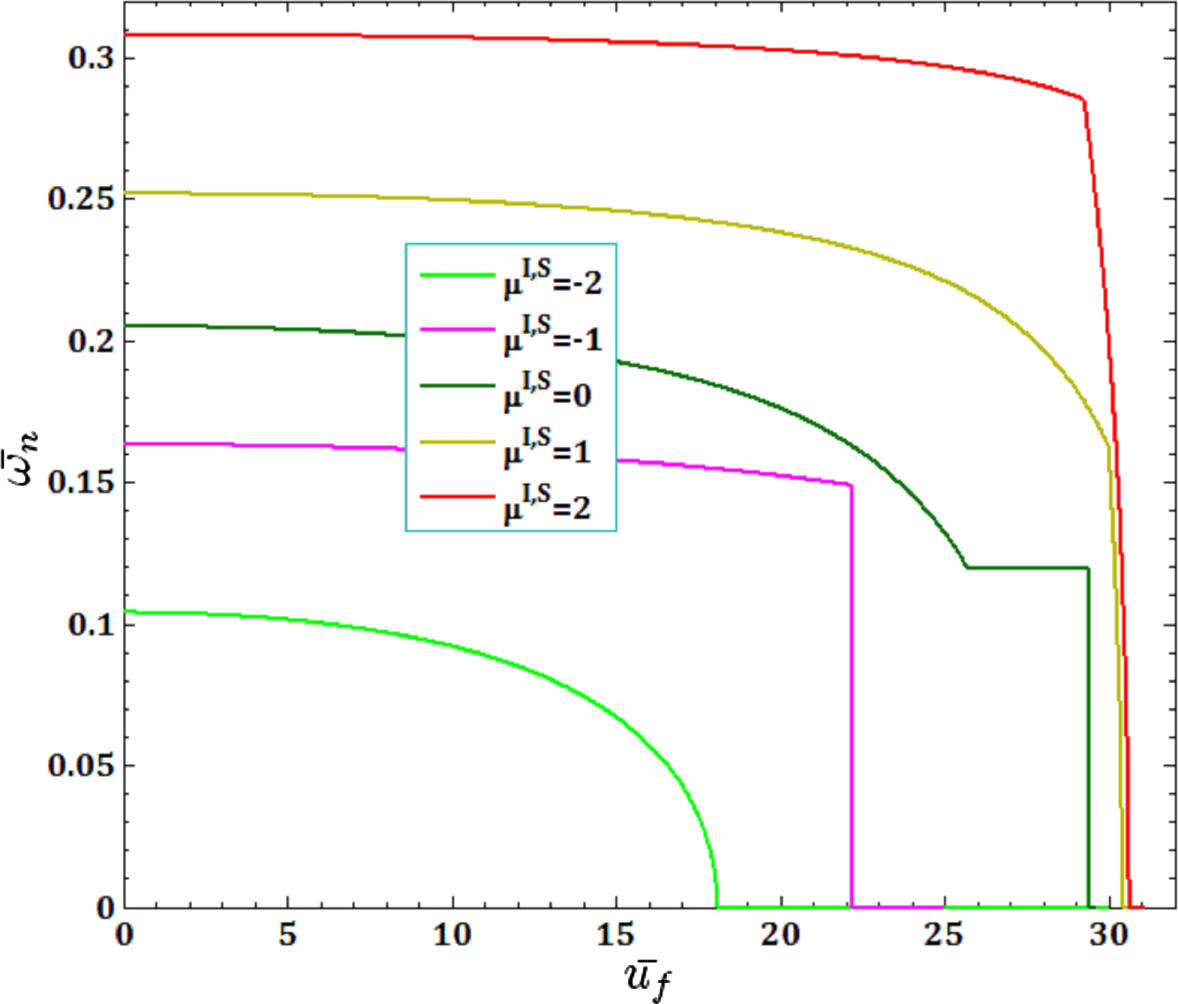
The effects of surface/interface Lame’s constants µ^I,S^ for fluid velocity 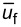 on DNF of SS FC-MWPENS.

**Figure 8:**
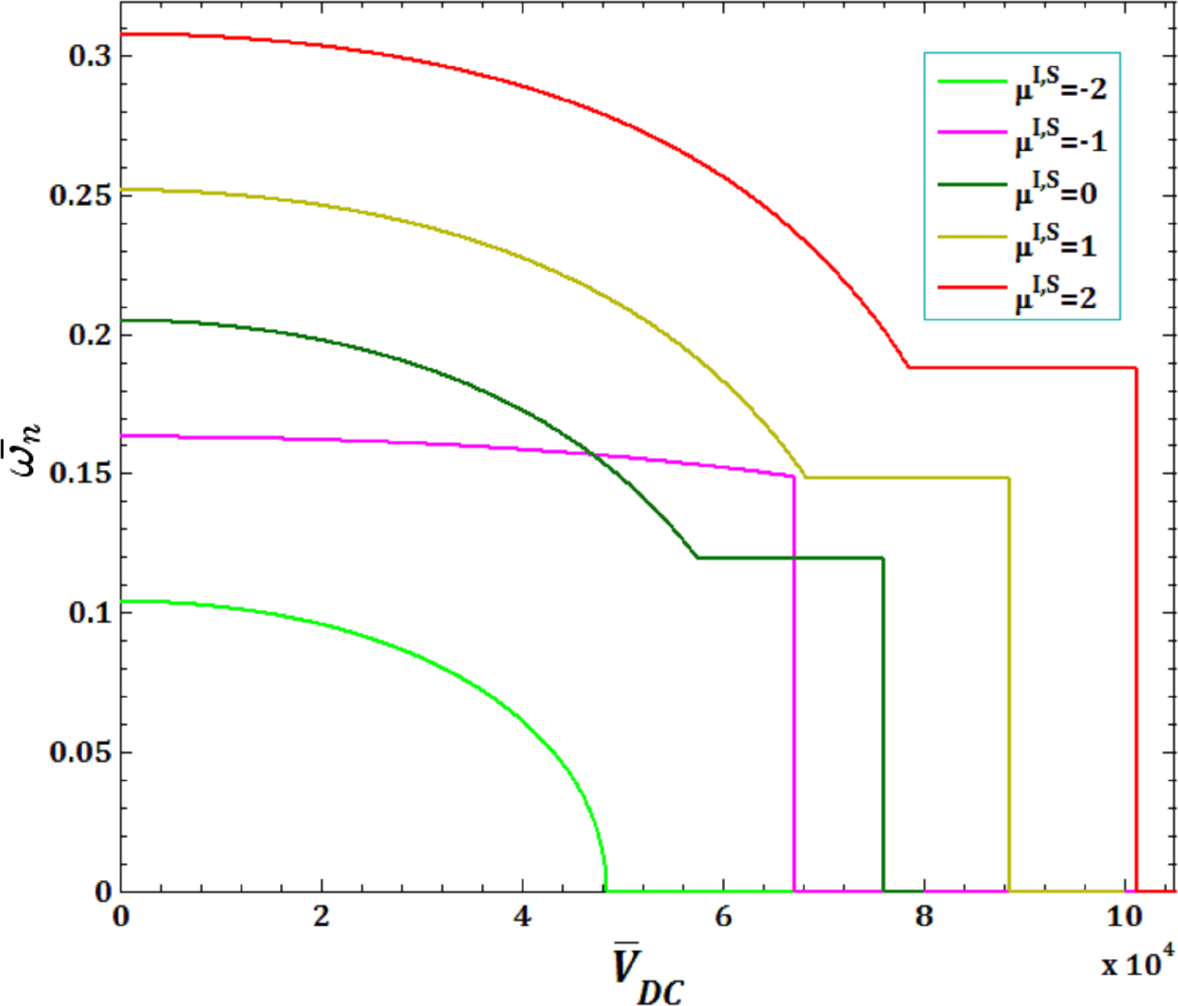
The effects of surface/interface Lame’s constants µ^I,S^ for pull-in voltage on DNF of SS FC-MWPENS.

The effects of different surface and interface Lame’s constants, 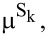 and 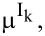 for viscous fluid velocity 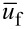 and pull-in instability analysis on DNF of FC-MWPENS are presented in Figure 7 and Figure 8. Similar to λ^I,S^, it is clear that by increasing both surface/interface Lame’s constants µ^I,S^, due to increasing FC-MWPENS stiffness, the DNF and also the critical fluid velocity and pull-in voltage increase.

**Figure 9:**
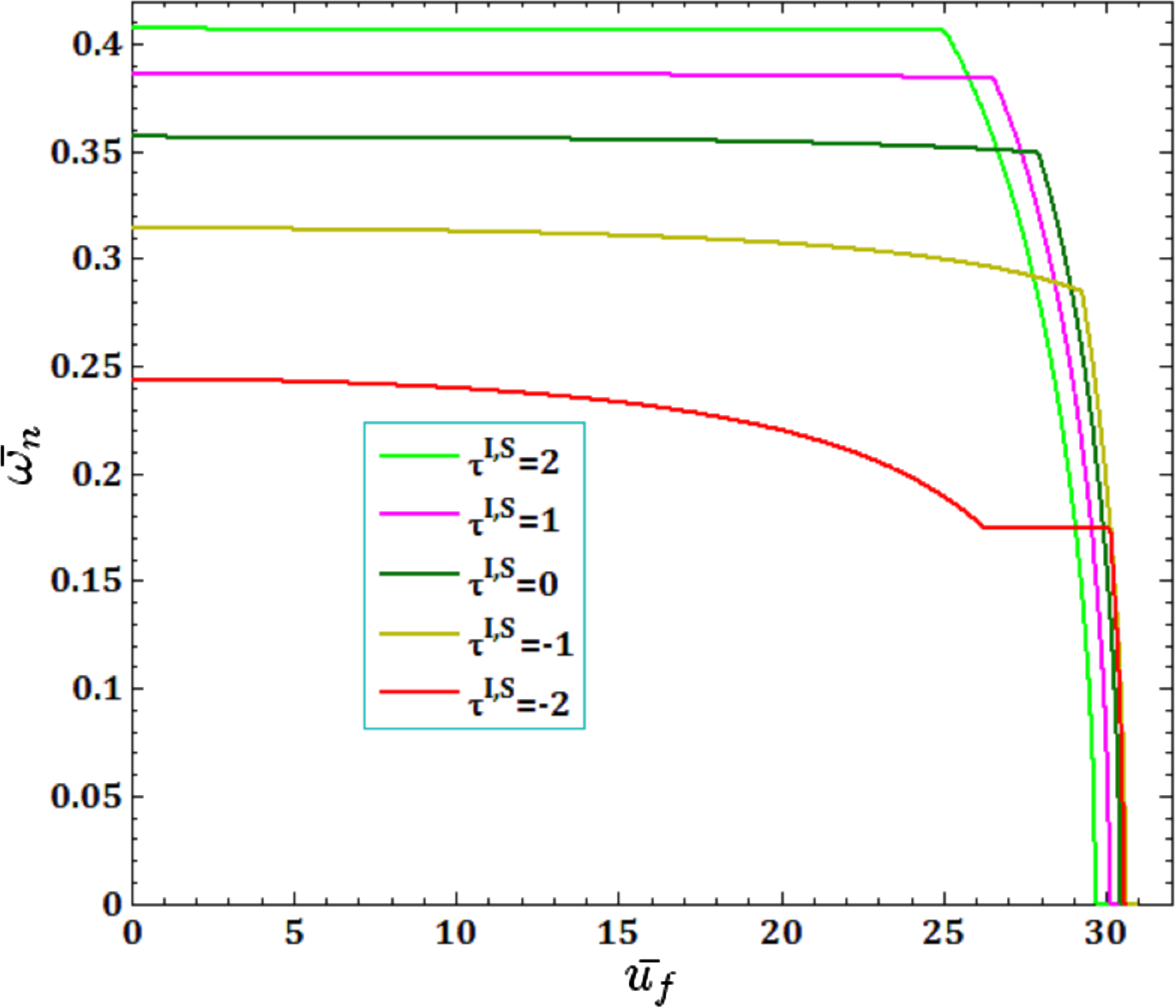
The effects of surface/interface residual stress 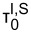 for fluid velocity 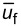 on DNF of SS FC-MWPENS.

**Figure 10:**
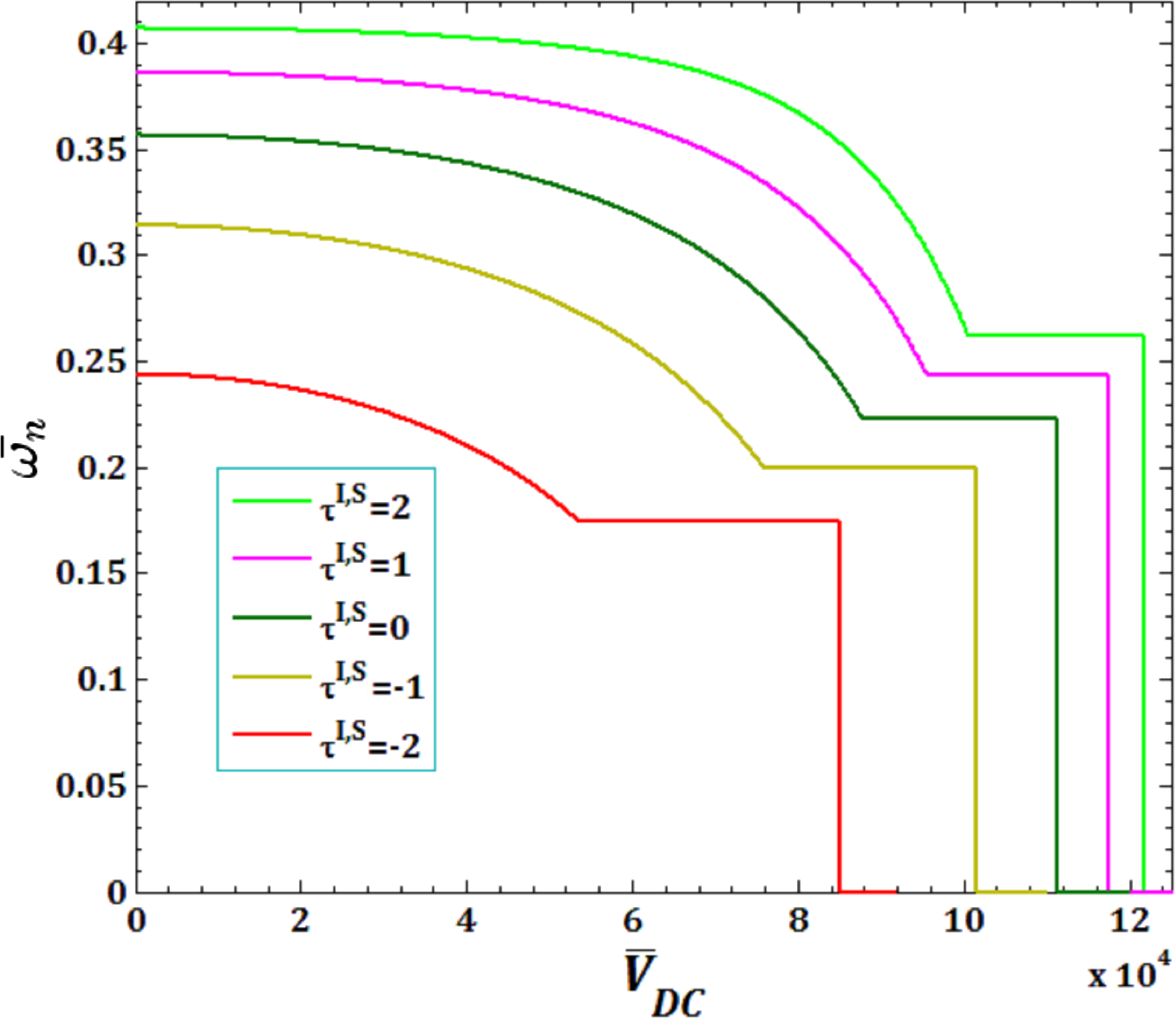
The effects of surface/interface residual stress 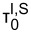 for pull-in voltage on DNF of SS FC-MWPENS.

Figure 9 and Figure 10 show the effects of surface and interface residual stress, 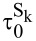 and 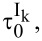 for viscous fluid velocity 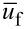 and pull-in instability analysis on DNF of FC-MWPENS. As can be seen in the analysis of DNF, increasing the surface/interface residual stress 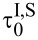 leads to increasing FC-MWPENS stiffness, and as a result, the DNF and pull-in voltage increase and critical fluid velocity decreases.

**Figure 11:**
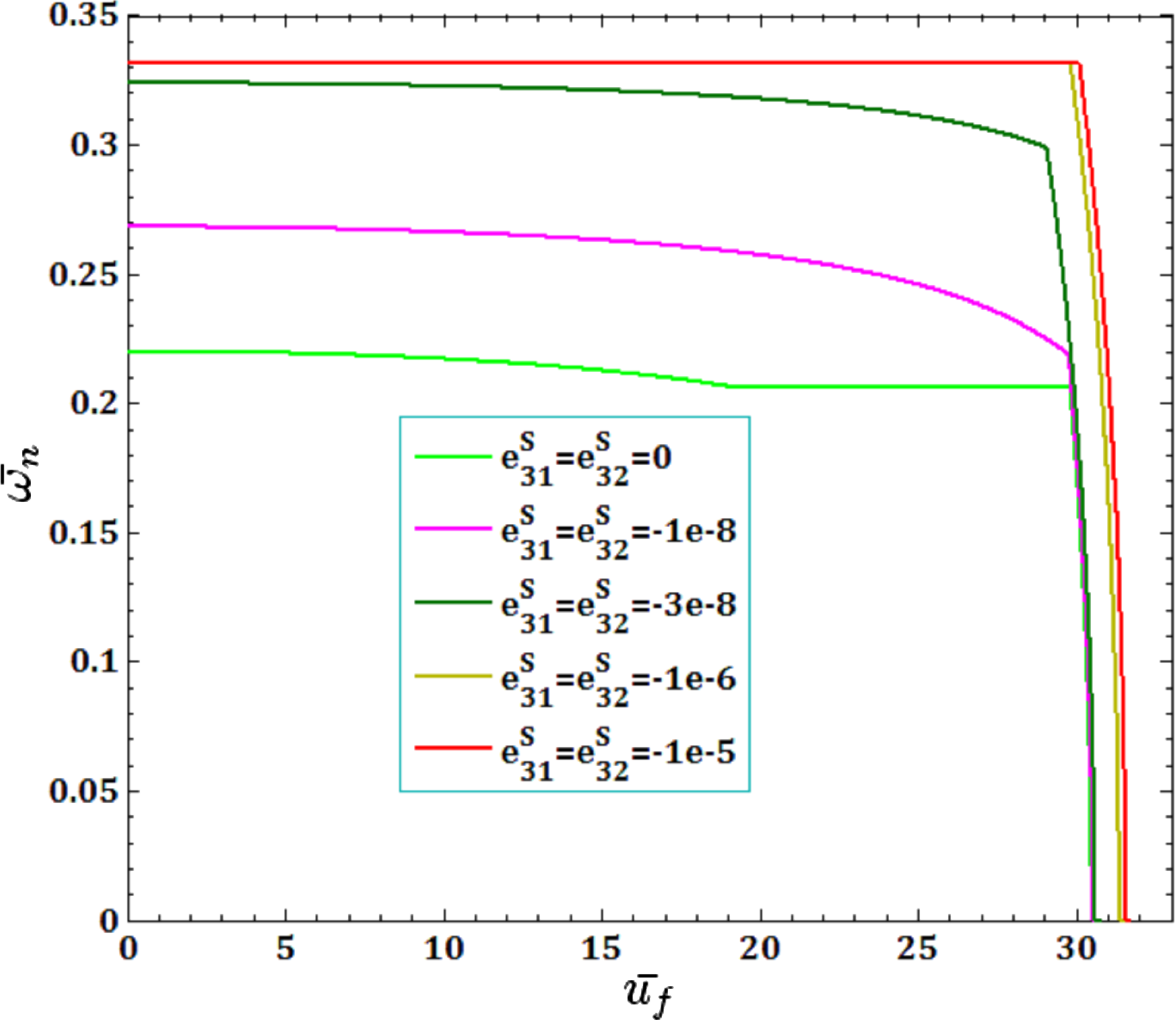
The effects of surface piezoelectricity constants 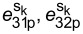 for fluid velocity 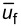 on DNF of SS FC-MWPENS.

**Figure 12:**
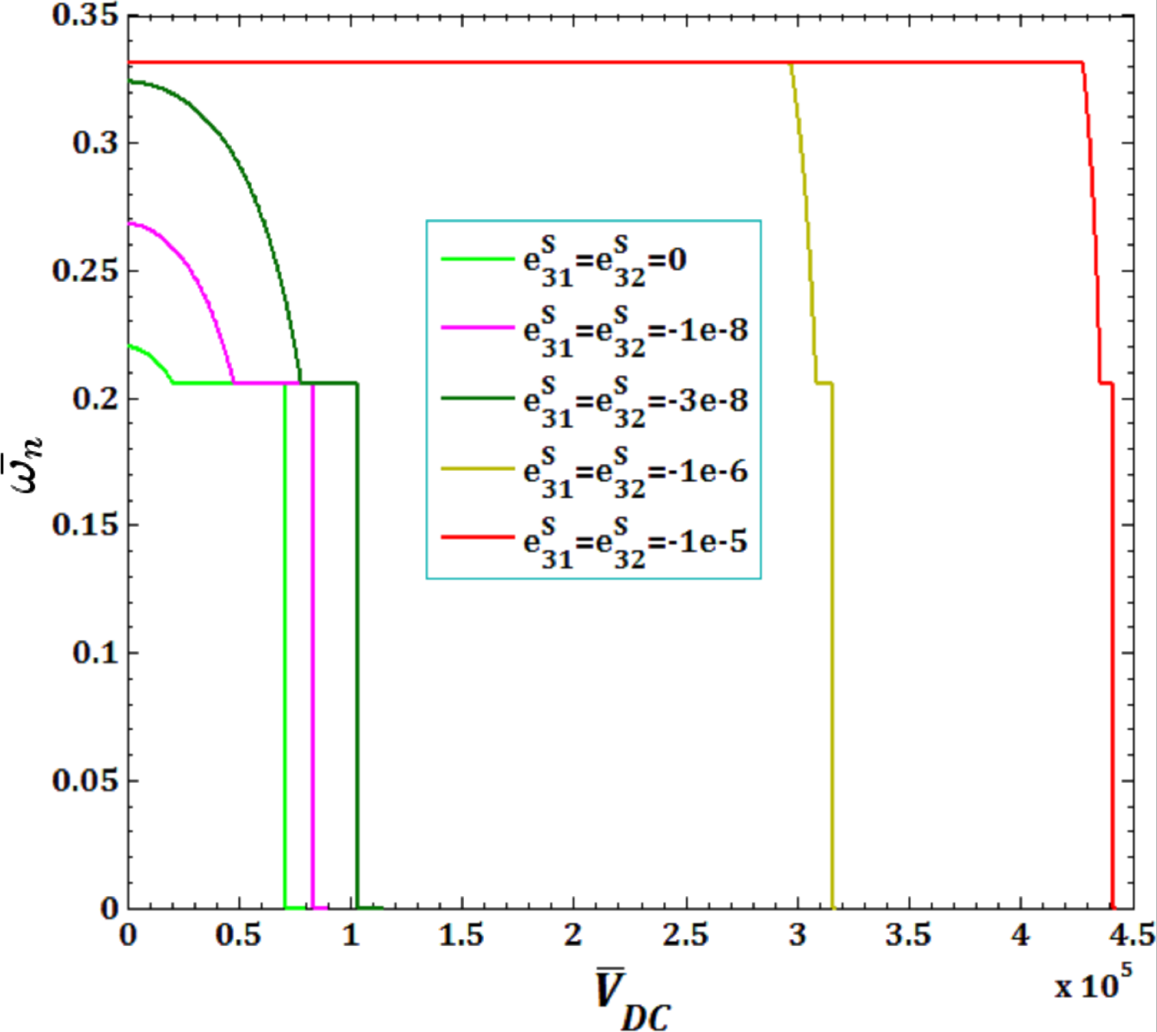
The effects of surface piezoelectricity constants 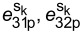 for pull-in voltage on DNF of SS FC-MWPENS.

The effect of surface piezoelectricity constants 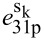 and 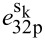 for viscous fluid velocity 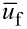 and pull-in instability analysis on DNF of FC-MWPENS is presented in Figure 11 and Figure 12. It is observed that the increase in the negative surface piezoelectricity constants 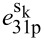 and 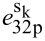 leads to increasing FC-MWPENS stiffness, and as a result, the DNF, critical fluid velocity and pull-in voltage increase.

**Figure 13:**
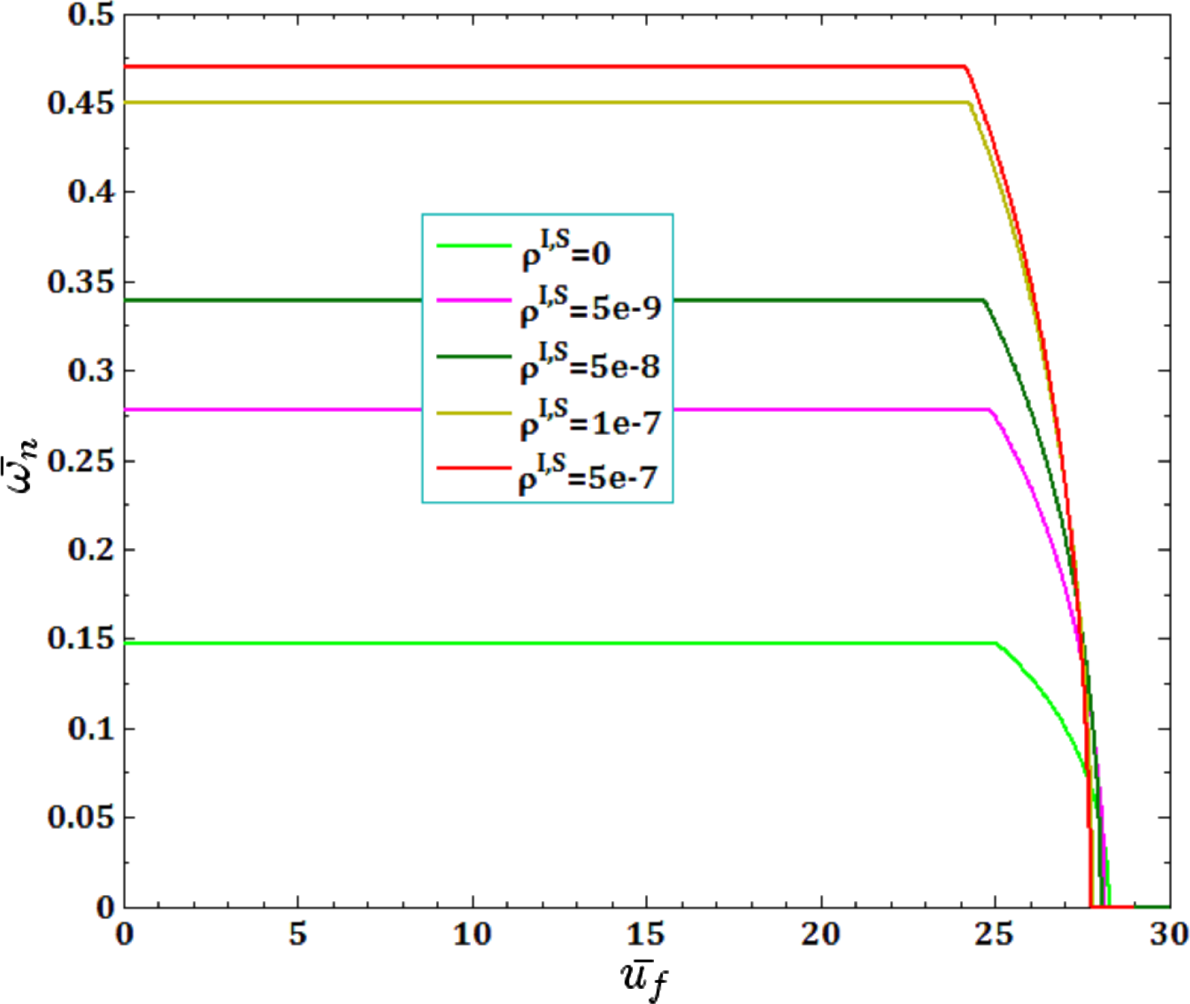
The effect of surface ρ^S^ and interface ρ^I^ mass density for fluid velocity 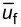 on DNF of SS FC-MWPENS.

**Figure 14:**
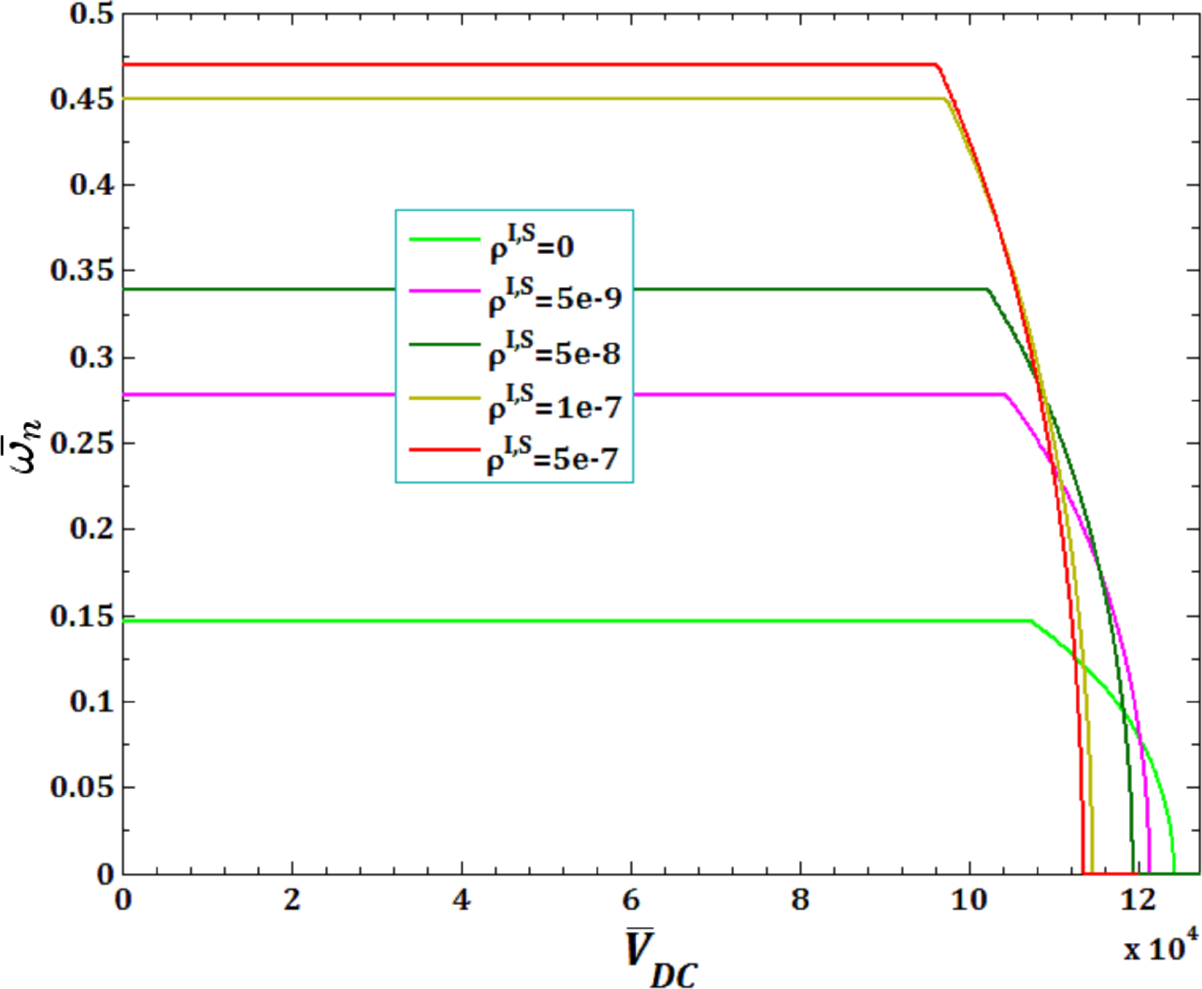
The effect of surface ρ^S^ and interface ρ^I^ mass density for pull-in voltage on DNF of SS FC-MWPENS.

Figure 13 and Figure 14 illustrate the effects of surface and interface mass density, 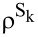 and 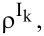 for viscous fluid velocity 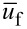 and pull-in instability analysis on DNF of FC-MWPENS. As it can be seen, with an increasing surface/interface mass density ρ^I,S^, due to increasing FC-MWPENS stiffness, the DNF significantly increases and also the critical fluid velocity and pull-in voltage slightly decrease.

**Figure 15:**
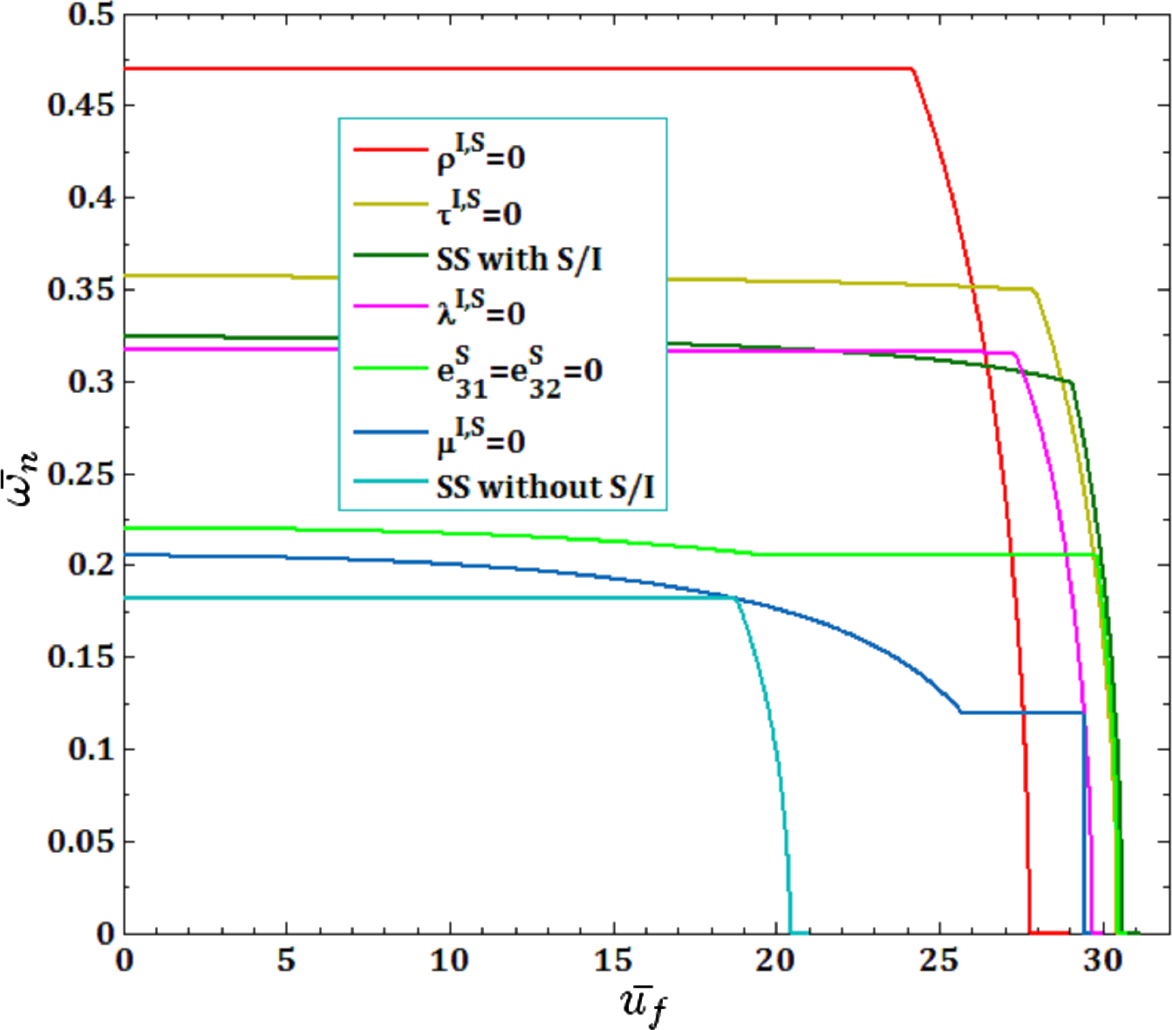
The effects of surface and interface parameters for fluid velocity 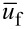 on DNF of SS FC-MWPENS.

**Figure 16:**
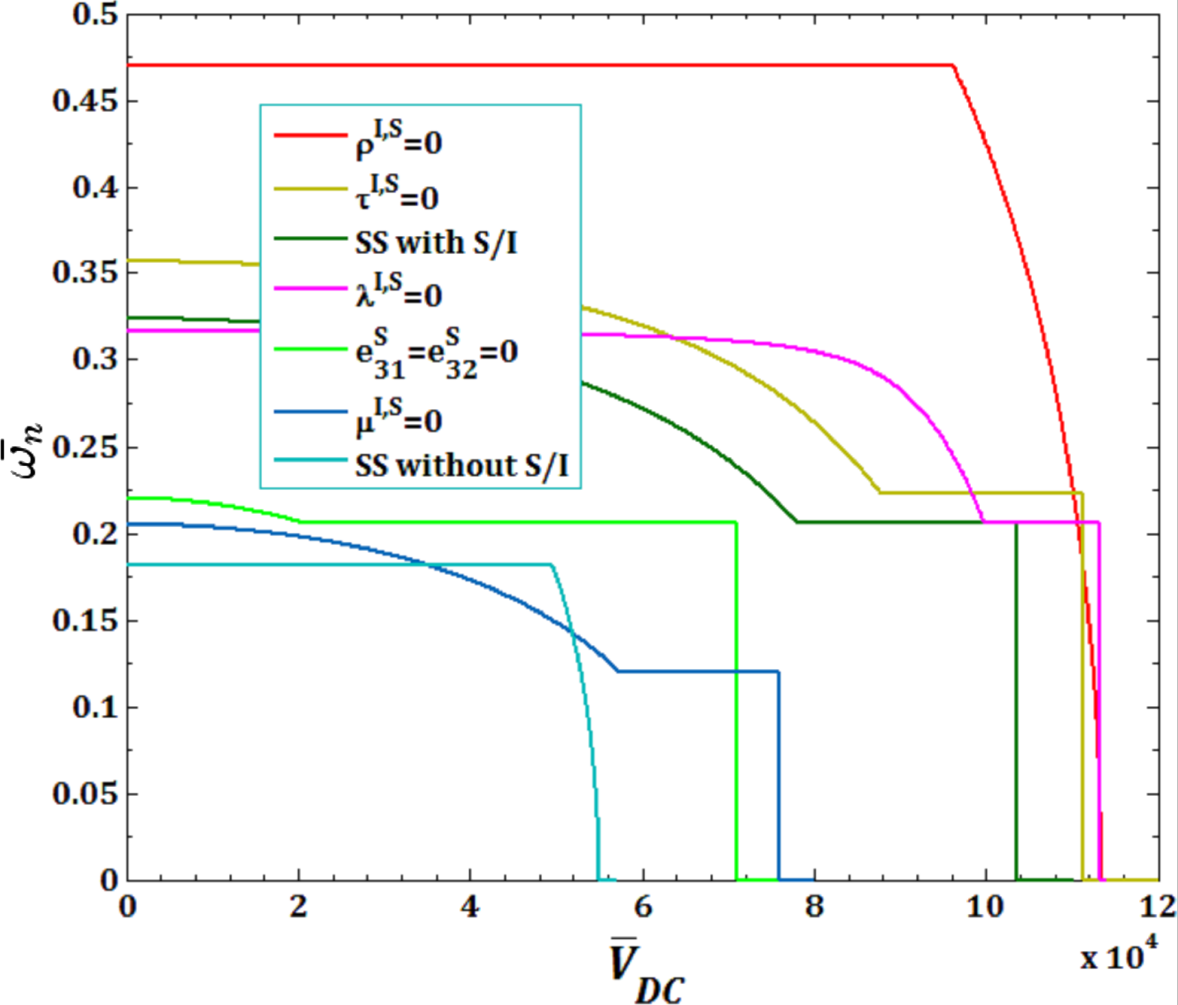
The effects of surface and interface parameters for pull-in voltage on DNF of SS FC-MWPENS.

In Figure 15 and Figure 16, the effects of all surface and interface parameters for viscous fluid velocity 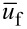 and pull-in instability analysis on DNF of SS FC-MWPENS are presented. It can be seen that by ignoring the surface/interface density ρ^I,S^, the inertia of the system will greatly decrease and due to increasing FC-MWPENS stiffness, the system will have a maximum DNF compared to other cases. Also when the surface/interface effects are not taken into account, due to the decreasing nanoshell stiffness, it has a lower DNF than the case with all surface/interface effects. In the cases without all surface/interface effects, the critical fluid velocity and also the pull-in voltage reach zero sooner than the rest of the parameters. In the cases without surface/interface density ρ^I,S^ and with all surface/interface effects, the pull-in voltage and critical fluid velocity reach zero later than the rest of the parameters and the system loses its stability due to the divergence via a pitchfork bifurcation.

## Conclusion

In the current study, the effect of the surface/interface parameters of a fluid-conveying multiwalled piezoelectric nanosensor are studied for analysis of the dimensionless natural frequency with respect to viscous fluid velocity 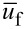 and pull-in voltage 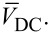 The piezoelectric nanosensor is simultaneously subjected to direct electrostatic DC voltage with nonlinear excitation, nonlinear van der Waals forces and a viscoelastic foundation. For this purpose, Hamilton’s principles, the assumed mode method combined with Lagrange–Euler’s equations are used. The results demonstrated that in the case of higher (lower) surface/interface densities, the inertia of the shell is increased (decreased) and its stiffness is reduced (increased), which leads to a decreasing (increasing) natural frequency compared to the case of without surface/interface effects. Also, by increasing both surface/interface Lame’s constants, λ^I,S^ and µ^I,S^, and the negative surface piezoelectricity constants, 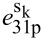 and 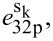 due to the increasing FC-MWPENS stiffness, the DNF and also the critical fluid velocity and pull-in voltage increase. In addition, in the analysis of DNF, it was found that increasing the surface/interface residual stress 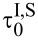 leads to increasing FC-MWPENS stiffness, and as a result, the DNF and pull-in voltage increase and critical fluid velocity decreases. Increasing the surface/interface mass density, ρ^I,S^, due to increasing FC-MWPENS stiffness, it was found that the DNF significantly increases and also the critical fluid velocity and pull-in voltage slightly decrease. Finally, by ignoring the surface/interface density, ρ^I,S^, the system will have a maximum DNF compared to other cases. In cases without all surface/interface effects, the critical fluid velocity and also the pull-in voltage reached zero sooner than the rest of the parameters. In the cases without surface/interface density ρ^I,S^ and with all surface/interface effects, the pull-in voltage and critical fluid velocity reach zero later than the rest of the parameters.

## Supporting Information

File 1 Subsections of “Mathematical Formulation” as well as an “Appendix” section.

File 2 MATLAB program code for the current paper.
In the ZIP file, all programs written in MATLAB software are presented for the results of the article, which is not included in the article due to the large volume of the program.
