# Supplementary material for: Vibration analysis and pull-in instability behavior in a multiwalled piezoelectric nanosensor with fluid flow conveyance
Source: Beilstein J Nanotechnol. 2020 Jul 21;11:1072–81. doi: 10.3762/bjnano.11.92 (PMC7385336; doi:10.3762/bjnano.11.92)
Supplement: File 1 — Subsections of “Mathematical Formulation” as well as an “Appendix” section. [file Beilstein_J_Nanotechnol-11-1072-s001.pdf]

BEILSTEIN JOURNAL OF NANOTECHNOLOGY

Supporting Information

for

Vibration analysis and pull-in instability behavior in a multiwalled piezoelectric nanosensor with fluid flow conveyance

Sayyid H. Hashemi Kachapi

Beilstein J. Nanotechnol. **2020,** *11,* 1072–1081. doi:10.3762/bjnano.11.92

Subsections of “Mathematical Formulation” as well as an “Appendix” section

License and Terms: This is a supporting information file under the terms of the Creative Commons Attribution License (http://creativecommons.org/licenses/by/4.0). Please note that the reuse, redistribution and reproduction in particular requires that the authors and source are credited.
The license is subject to the *Beilstein Journal of Nanotechnology* terms and conditions: (https://www.beilstein-journals.org/bjnano)

Mathematical Formulation

Non-classical Shell theory

Within the framework of Gurtin–Murdoch surface/interface elasticity theory, the normal stresses σ_{xx} and $\sigma_{\theta\theta}$ can be written as [1,2-5]

$$\sigma_{xx(N,p)} = C_{11(N,p)}\varepsilon_{xx} + C_{12(N,p)}\varepsilon_{\theta\theta} - e_{31p}\bar{E}_{xp} + \frac{\upsilon_{(N,p)}\sigma_{zz(N,p)}}{1-\upsilon_{(N,p)}}, \quad \text{(S1)}$$

$$\sigma_{\theta\theta(N,p)} = C_{21(N,p)}\varepsilon_{xx} + C_{22(N,p)}\varepsilon_{\theta\theta} - e_{32p}\bar{E}_{\theta p} + \frac{\upsilon_{(N,p)}\sigma_{zz(N,p)}}{1-\upsilon_{(N,p)}}, \quad \text{(S2)}$$

$$\sigma_{x\theta(N,p)} = C_{66(N,p)}\gamma_{x\theta}, \quad \text{(S3)}$$

where based on nonclassical continuum model, σ_{zz} is expressed as following

$$\sigma_{zz} = \frac{z}{h_{Nn}+h_{p2}}\left((\tau_0^{ps}+\tau_0^{NI})(\frac{\partial^2 w}{\partial x^2}+\frac{1}{R^2}\frac{\partial^2 w}{\partial \theta^2}) - (\rho^{ps}+\rho^{NI})\frac{\partial^2 w}{\partial t^2}\right), \quad \text{(S4)}$$

In all following formulations, all of the piezoelectric parameters (the materials and geometrical parameters) are neglected for first layer and to be zero and all of the materials and geometrical parameters of nanoshell in the first layer are similar to the second layer of nanostructure.

Also all coefficients and phrases of Equations S1–S4 such as nonlinear deflection, displacement fields and curvatures, relations of Gurtin–Murdoch surface/interface elasticity theory and etc. can be expressed in full detail in [3-5].

Governing equations

In current section, the governing equations and boundary conditions of the MW piezoelectric nanostructure are obtained by using the Hamilton principle. The total strain energy considering the surface/interface effect is written as:

$$\begin{aligned}\pi_n &= \frac{1}{2}\int_0^L\int_0^{2\pi}\int_{-h_N}^{h_N}\sigma_{ijN}\varepsilon_{ij}\,Rdzd\theta dx + \frac{1}{2}\int_0^L\int_0^{2\pi}\int_{h_N}^{h_N+h_p}\left(\sigma_{ijp}\varepsilon_{ij} - \bar{E}_{zp2}D_{zp}\right)Rdzd\theta dx \\ &+ \frac{1}{2}\int_0^L\int_0^{2\pi}\left(\sigma_{ij}^{s_2}\varepsilon_{ij} - \bar{E}_{zp}D_i^{s_2}\right)(R+h_N+h_p)\,d\theta dx + \frac{1}{2}\int_0^L\int_0^{2\pi}\sigma_{ij}^{s_1}\varepsilon_{ij}\,(R-h_N)d\theta dx \\ &= \frac{1}{2}\int_0^L\int_0^{2\pi}\left\{\begin{matrix} N_{xxn}\varepsilon_{xx}^0 + N_{\theta\theta n}\varepsilon_{\theta\theta}^0 + N_{x\theta n}\gamma_{x\theta}^0 + M_{xxn}\kappa_{xx} + M_{\theta\theta n}\kappa_{\theta\theta} + M_{x\theta n}\kappa_{x\theta} \\ +\eta_{33}\bar{E}_{zp}^2 h_p \end{matrix}\right\}R_n d\theta dx\end{aligned} \quad \text{(S5)}$$

In Equation S5, the forces (N) and moment (M) resultants are defined in reference Hashemi Kachapi et al. [5] for SWPENS. And, as explained, all the sentences mentioned in [5] belong to

the last wall (in this article, the third wall), which is considered a nanoshell with two piezoelectric layers, and in the other walls (in this article, the first and second walls) are not considered piezoelectric effects. The kinetic energy of the FC-MWPENS can be written as:

$$T_n = \frac{1}{2}\iint I_n\left(\left(\frac{\partial u_n}{\partial t}\right)^2 + \left(\frac{\partial v_n}{\partial t}\right)^2 + \left(\frac{\partial w_n}{\partial t}\right)^2\right) R_n d\theta dx \tag{S6}$$

where

$$I_n = \int_{-h_N}^{h_N} \rho_N \, dz + \int_{-h_N-h_p}^{-h_N} \rho_p \, dz + \int_{h_N}^{h_N+h_p} \rho_p \, dz + \rho^{S,I}$$
$$= 2\rho_N h_N + 2\rho_p h_p + 2\rho^{ps} + 2\rho^{NI}$$

The work done by the surrounded viscoelastic medium including the viscoelastic foundation and also the nonlinear van der Waals interaction and the nonlinear electrostatic force for example for three walled piezoelectric nano-sensor (TWPENS), respectively, can be expressed as [5-7]

$$W_{vdw} = \int_0^L \int_0^{2\pi} \int_0^{w_1} \left(C_{vdw(12)}^{L}(w_2 - w_1) + C_{vdw(12)}^{NL}(w_2 - w_1)^3\right) dw_1 \, R_1 d\theta dx$$
$$+ \int_0^L \int_0^{2\pi} \int_0^{w_2} \begin{pmatrix} C_{vdw(23)}^{L}(w_3 - w_2) + C_{vdw(23)}^{NL}(w_3 - w_2)^3 \\ -\left(\frac{R_1}{R_2}\right)\left(C_{vdw(12)}^{L}(w_2 - w_1) + C_{vdw(12)}^{NL}(w_2 - w_1)^3\right) \end{pmatrix} dw_2 \, R_2 d\theta dx \tag{S7}$$
$$- \int_0^L \int_0^{2\pi} \int_0^{w_3} \left(\frac{R_2}{R_3}\right)\left(C_{vdw(32)}^{L}(w_3 - w_2) + C_{vdw(32)}^{NL}(w_3 - w_2)^3\right) dw_3 \, R_3 d\theta dx,$$

$$W_{vm} = -\int_0^L \int_0^{2\pi} \int_0^{w_3} \left(K_w w_3 - K_p \nabla^2 w_3 + C_w \frac{\partial w_3}{\partial t}\right) dw_3 R_3 d\theta dx, \tag{S8}$$

$$W_e = \int_0^L \int_0^{2\pi} \int_0^{w_3} \frac{\pi \Upsilon (V_{DC} + V_{AC}\cos(\omega t))^2}{\sqrt{(b_2 - w_3)(2R_3 + b_3 - w_3)}\left[\cosh^{-1}\left(1 + \frac{b_3 - w_3}{R_3}\right)\right]^2} dw_3 \, R_3 d\theta dx, \tag{S9}$$

where all coefficients and phrases of Equations S7–S9 can be seen in [3-5]. Also, the external work of the fluid can be written as [8]

$$W_f = \frac{1}{2}\int_0^L \int_0^{2\pi} F_{fluid} \, w \, R d\theta dx \tag{S10}$$

$$
=\frac{1}{2}\int_0^L\int_0^{2\pi}\left\{\begin{array}{c}-\rho_f A_f\left(\begin{array}{c}\frac{\partial^2 w}{\partial t^2}+2\left(VCF\times V_{no-slip}\right)\frac{\partial^2 w}{\partial x\partial t}\\+\left(VCF\times V_{no-slip}\right)^2\frac{\partial^2 w}{\partial x^2}\end{array}\right)\\+\mu A_f\left(\begin{array}{c}\frac{\partial^3 w}{\partial x^2\partial t}+\frac{\partial^3 w}{R^2\partial\theta^2\partial t}\\+\left(VCF\times V_{no-slip}\right)\left(\frac{\partial^3 w}{\partial x^3}+\frac{\partial^3 w}{R^2\partial x\partial\theta^2}\right)\end{array}\right)\end{array}\right\}w\,Rd\theta dx
$$

By applying of following Hamilton's principle

$$
\int_0^t\left(\delta T_n-\delta\pi_n+\delta w_{vm}+\delta w_{vdw}+\delta w_e+\delta w_f\right)dt=0, \tag{S11}
$$

and substituting Equations S5–S10 into Equation S11, the governing equations of motion and boundary conditions for FC-TWPENS respectively are obtained as follows:

$$
\delta u_n:\quad \frac{\partial N_{xn}}{\partial x}+\frac{1}{R_n}\frac{\partial N_{x\theta n}}{\partial\theta}=I_n\frac{\partial^2 u_n}{\partial t^2}, \tag{S12}
$$

$$
\delta v_n:\quad \frac{\partial N_{x\theta n}}{\partial x}+\frac{1}{R_n}\frac{\partial N_{\theta n}}{\partial\theta}=I_n\frac{\partial^2 v_n}{\partial t^2}, \tag{S13}
$$

$$
\begin{aligned}
\delta w_n:\quad &\frac{\partial^2 M_{xn}}{\partial x^2}+\frac{2}{R_n}\frac{\partial^2 M_{x\theta n}}{\partial x\partial\theta}+\frac{1}{R_n^2}\frac{\partial^2 M_{\theta n}}{\partial\theta^2}-\frac{N_{\theta n}}{R_n}+N_{xn}\frac{\partial^2 w_n}{\partial x^2}+\frac{\partial N_{xn}}{\partial x}\frac{\partial w_n}{\partial x}\\
&+\frac{N_{\theta n}}{R_n^2}\frac{\partial^2 w_n}{\partial\theta^2}\\
&+\frac{1}{R_n^2}\frac{\partial N_{\theta n}}{\partial\theta}\frac{\partial w_n}{\partial\theta}+\frac{2}{R_n}N_{x\theta n}\frac{\partial^2 w_n}{\partial x\partial\theta}+\frac{1}{R_n}\frac{\partial N_{x\theta n}}{\partial x}\frac{\partial w_n}{\partial\theta}+\frac{1}{R_n}\frac{\partial N_{x\theta n}}{\partial\theta}\frac{\partial w_n}{\partial x}=I_n\frac{\partial^2 w_n}{\partial t^2}+S_n\\
&-\frac{\pi\Upsilon(V_{DC}+V_{AC}\cos(\omega t))^2}{\sqrt{(b_2-w_2)(2R_2+b_2-w_2)}\left[\cosh^{-1}\left(1+\frac{b_2-w_2}{R_2}\right)\right]^2},
\end{aligned} \tag{S14}
$$

where S_n for the inner and outer layer, respectively, are:

$$
S_1=-\left(C^L_{vdw(12)}(w_2-w_1)+C^{NL}_{vdw(12)}(w_2-w_1)^3\right),) \tag{S15}
$$

$$
S_2=\left(\begin{array}{c}-C^L_{vdw(23)}(w_3-w_2)-C^{NL}_{vdw(23)}(w_3-w_2)^3\\+\left(\frac{R_1}{R_2}\right)\left(C^L_{vdw(12)}(w_2-w_1)+C^{NL}_{vdw(12)}(w_2-w_1)^3\right)\end{array}\right) \tag{S16}
$$

$$S_3 = \begin{pmatrix} \left(\frac{R_2}{R_3}\right)\left(C_{vdw(32)}^{L}(w_3 - w_2) + C_{vdw(32)}^{NL}(w_3 - w_2)^3\right) \\ +K_w w_3 - K_p \nabla^2 w_3 + C_w \frac{\partial w_3}{\partial t} \end{pmatrix} \tag{S17}$$

and boundary conditions are obtained as follows:

$$\delta u_n = 0 \quad or \qquad N_{xn} n_x + \frac{1}{R_n} N_{x\theta n} n_\theta = 0, \tag{S18}$$

$$\delta v_n = 0 \quad or \qquad N_{x\theta n} n_x + \frac{1}{R_n} N_{\theta n} n_\theta = 0, \tag{S19}$$

$$\begin{aligned} \delta w_n = 0 \quad or \quad & \left(\frac{\partial M_{xn}}{\partial x} + \frac{1}{R_n}\frac{\partial M_{x\theta n}}{\partial \theta} + N_{xx}\frac{\partial w_n}{\partial x} + \frac{N_{x\theta}}{R_n}\frac{\partial w_n}{\partial \theta}\right) n_x \\ + \left(\frac{1}{R_n}\frac{\partial M_{x\theta n}}{\partial x} + \frac{1}{R_n^2}\frac{\partial M_{\theta n}}{\partial \theta} + \frac{N_{x\theta n}}{R_n}\frac{\partial w_n}{\partial x} + \frac{N_{\theta n}}{R_n^2}\frac{\partial w_n}{\partial \theta}\right) n_\theta = 0, \end{aligned} \tag{S20}$$

$$\frac{\partial w_n}{\partial x} = 0 \quad or \qquad M_{xn} n_x + \frac{1}{R_n} M_{x\theta n} n_\theta = 0, \tag{S21}$$

$$\frac{\partial w_n}{\partial \theta} = 0 \quad or \qquad \frac{1}{R_n} M_{x\theta n} n_x + \frac{1}{R_n^2} M_{\theta n} n_\theta = 0, \tag{S22}$$

The following dimensional parameters are also used to dimensionless equations of motion and boundary conditions.

$$\begin{aligned} &\bar{u}_n = \frac{u_n}{h_{Nn}}, \bar{v}_n = \frac{v_n}{h_{Nn}}, \bar{w}_n = \frac{w_n}{h_{Nn}}, \xi_n = \frac{x_n}{L}, \bar{b}_n = \frac{b_n}{L}, \bar{A}_{ijNn} = \frac{A_{ijNn}}{A_{11Nn}}, \bar{B}_{ijNn} = \frac{B_{ijNn}}{A_{11Nn} h_{Nn}}, \\ &\bar{D}_{ijNn} = \frac{D_{ijNn}}{A_{11Nn} h_{Nn}^2}, \bar{A}_{ijpn} = \frac{A_{ijpn}}{A_{11Nn}}, \bar{A}_{ijn}^* = \frac{A_{ijn}^*}{A_{11Nn}}, \bar{B}_{ijpn} = \frac{B_{ijpn}}{A_{11Nn} h_{Nn}}, \bar{B}_{ijn}^* = \frac{B_{ijn}^*}{A_{11Nn} h_{Nn}}, \\ &\bar{D}_{ijpn} = \frac{D_{ijpn}}{A_{11Nn} h_{Nn}^2}, \bar{D}_{ijn}^* = \frac{D_{ijn}^*}{A_{11Nn} h_{Nn}^2}, \bar{F}_{11Nn}^* = \frac{F_{11Nn}^*}{A_{11Nn} h_{Nn}}, \bar{F}_{11pn}^* = \frac{F_{11pn}^*}{A_{11Nn} h_{Nn}}, \\ &\bar{E}_{11Nn}^* = \frac{E_{11Nn}^*}{A_{11Nn} h_{Nn}^2}, \bar{E}_{11pn}^* = \frac{E_{11pn}^*}{A_{11Nn} h_{Nn}^2}, \bar{J}_{11Nn}^* = \frac{J_{11Nn}^*}{\rho_{Nn} h_{Nn}^2}, J_{11pn}^* = \frac{J_{11pn}^*}{\rho_{Nn} h_{Nn}^2}, \\ &\bar{G}_{11Nn}^* = \frac{G_{11Nn}^*}{\rho_{Nn} h_{Nn}^3}, G_{11pn}^* = \frac{G_{11pn}^*}{\rho_{Nn} h_{Nn}^3}, \bar{N}_{xpn}^* = \frac{N_{xpn}^* V_0}{A_{11Nn}}, \bar{N}_{\theta pn}^* = \frac{N_{\theta pn}^* V_0}{A_{11Nn}}, \bar{M}_{xpn}^* = \frac{M_{xpn}^* V_0}{A_{11Nn} h_{Nn}}, \end{aligned} \tag{S23}$$

$$\bar{M}^*_{\theta pn} = \frac{M^*_{\theta pn} V_0}{A_{11Nn} h_{Nn}}, \bar{\tau}_0^{sn} = \frac{\tau_0^{sn}}{A_{11Nn}}, \bar{R}_n = \frac{R_n}{L}, m_{0n} = \frac{L}{R_n}, m_{1n} = \frac{L}{h_{Nn}}, m_{2n} = \frac{h_{Nn}}{R_n} = \bar{h}_{Nn},$$

$$\bar{h}_{pn} = \frac{h_{pn}}{R_n}, m_{3n} = \frac{I_n}{2\rho_{Nn} h_{Nn}}, m_{4n} = \frac{h_{pn}}{h_{Nn}}, \Omega_{\mathrm{n}} = \sqrt{\frac{A_{11Nn}}{2\rho_{Nn} h_{Nn} L^2}}, \tau_n = \Omega_{\mathrm{n}} t_n, \bar{\omega}_n = \frac{\omega_n}{\Omega_{\mathrm{n}}},$$

$$\bar{K}_w = \frac{K_w L^2}{m_3 A_{11N}}, \bar{K}_p = \frac{K_p}{m_3 A_{11N}}, \bar{C}_{w2} = \frac{C_w \Omega L^2}{m_{3n} A_{11Nn}}, \bar{C}^{L}_{vdwn} = \frac{C^{L}_{vdwn} L^2}{m_{3n} A_{11Nn}},$$

$$\bar{C}^{NL}_{vdwn} = \frac{C^{NL}_{vdwn} L^2 h_{Nn}^2}{m_{3n} A_{11Nn}}, \bar{\rho}_f = \frac{\rho_f}{m_3 \rho_N}, \bar{u}_f = \left(VCF \times V_{no-slip}\right)\sqrt{\frac{2\rho_N h_N}{A_{11N}}},$$

$$\bar{\mu}_f = \frac{\mu_f}{m_3}\sqrt{\frac{2h_N}{\rho_N A_{11N} L^2}}, \bar{V}_{DC} = \frac{V_{DC}}{V_0}, \bar{V}_{p2} = \frac{V_{p2}}{V_0}, \bar{F}_e = \frac{\pi m_1^2 V_0^2 \Upsilon}{m_3 A_{11N}},$$

In current study, the electrostatic force Equation S9 can be expressed as a polynomial form that is solved by nonlinear curve-fitting problem of lsqcurvefit function in Matlab Toolbox using least-squares. Therefore, the dimensionless electrostatic work can be written as follows [5]:

$$W_e = \int_0^L \int_0^{2\pi} \int_0^{\bar{w}_3} \bar{F}_e(\bar{V}_{DC} + \bar{V}_{AC}\cos(\bar{\omega}\tau))^2 \begin{pmatrix} \bar{C}_1 + \bar{C}_2 \bar{w}_3 + \bar{C}_3 \bar{w}_3^2 \\ + \cdots + \bar{C}_n \bar{w}_3^{n-1} \end{pmatrix} d\bar{w}_3\, \bar{R}_3 d\theta d\xi \qquad \text{(S24)}$$

Solution procedure

By using the following shear deformation and displacement in the assumed mode method [3-5]

$$\begin{bmatrix} u_n(x,\theta,t) \\ v_n(x,\theta,t) \\ w_n(x,\theta,t) \end{bmatrix} = \sum_{m=1}^{M_1}\sum_{j=1}^{N} \begin{bmatrix} \left[u_{m,j,c}(\tau)\cos(j\theta) + u_{m,j,s}(\tau)\sin(j\theta)\right]\chi_{mj}(\xi) \\ \left[v_{m,j,c}(\tau)\sin(j\theta) + v_{m,j,s}(\tau)\cos(j\theta)\right]\phi_{mj}(\xi) \\ \left[w_{m,j,c}(\tau)\cos(j\theta) + w_{m,j,s}(\tau)\sin(j\theta)\right]\beta_{mj}(\xi) \end{bmatrix} + \sum_{m=1}^{M_2} \begin{bmatrix} u_{m,0}(\tau)\chi_{m0}(\xi) \\ v_{m,0}(\tau)\phi_{m0}(\xi) \\ w_{m,0}(\tau)\beta_{m0}(\xi) \end{bmatrix} = \sum_{(i,r,s)=1}^{M_2+M_1\times N} \begin{bmatrix} u_{ni}(\tau)\chi_{ni}(\xi)\vartheta_{ni}(\theta) \\ v_{nr}(\tau)\phi_{nr}(\xi)\alpha_{nr}(\theta) \\ w_{ns}(\tau)\beta_{ns}(\xi)\psi_{ns}(\theta) \end{bmatrix}, \qquad \text{(S25)}$$

And using dimensionless strain and kinetic energies Equations S5 and S6 and dimensionless applied works Equations S7–S10 and substituting of the Lagrange–Euler equations, the following reduced-order model of the nonlinear equations of motion are obtained:

$$[(M)_u^u]_n\{\ddot{\bar{u}}_n\} + [(M)_u^w]_n\{\ddot{\bar{w}}_n\} + [(K)_u^u]_n\{\bar{u}_n\} + [(K)_u^v]_n\{\bar{v}_n\} + [(K)_u^w]_n\{\bar{w}_n\} + [(NL)_u^w]_n\{\bar{w}_n^2\} = \bar{F}_{un}, \qquad \text{(S26)}$$

$$[(M)_v^v]_n\{\ddot{\bar{v}}_n\} + [(M)_v^w]_n\{\ddot{\bar{w}}_n\} + [(K)_v^v]_n\{\bar{v}_n\} + [(K)_v^u]_n\{\bar{u}_n\} + [(K)_v^w]_n\{\bar{w}_n\}$$
$$+[(NL)_v^w]_n]\{\bar{w}_n^2\} = \bar{F}_{vn}, \tag{S27}$$

$$\left[[(M)_w^w]_n + \left[(K)_{w2}^{\ddot{w}}\right]_n\{\bar{w}_n\}\right]\{\ddot{\bar{w}}_n\} + [(c)_w^w]\{\dot{\bar{w}}_n\} + [(K)_w^u]_n\{\bar{u}_n\} + [(K)_w^v]_n\{\bar{v}_n\} \tag{S28}$$
$$+[[(K)_w^w]_n - \bar{F}_{e2}(K_e)_w^w]\{\bar{w}_n\}$$
$$+(-1)^n q_1\left(\frac{\bar{R}_{n-1}}{\bar{R}_n}\right)^{m_1} \bar{C}_{vdw((n-1)n)}^{\,L}\left([(K)_{w1n}^{vdw}]\{\bar{w}_n\} - [(K)_{w2n}^{vdw}]\{\bar{w}_{n-1}\}\right)$$
$$+(-1)^n q_2\left(\frac{\bar{R}_n}{\bar{R}_{n+1}}\right)^{m_2} \bar{C}_{vdw(n(n+1))}^{\,L}\left([(K)_{w3n}^{vdw}]\{\bar{w}_{n+1}\} - [(K)_{w4n}^{vdw}]\{\bar{w}_n\}\right)$$
$$+(-1)^n q_1\left(\frac{\bar{R}_{n-1}}{\bar{R}_n}\right)^{m_1}\left(\bar{C}_{vdw((n-1)n)}^{\,NL}\right)\begin{pmatrix}[(NL)_{w1n}^{vdw}]\bar{w}_n^3 - 3[(NL)_{w2n}^{vdw}]\bar{w}_n^2\bar{w}_{n-1}\\ +3[(NL)_{w3n}^{vdw}]\bar{w}_n\bar{w}_{n-1}^2 - [(NL)_{w4n}^{vdw}]\bar{w}_{n-1}^3\end{pmatrix}$$
$$+(-1)^n q_2\left(\frac{\bar{R}_n}{\bar{R}_{n+1}}\right)^{m_2}\left(\bar{C}_{vdw(n(n+1))}^{\,NL}\right)\begin{pmatrix}[(NL)_{w5n}^{vdw}]\bar{w}_{n+1}^3 - 3[(NL)_{w6n}^{vdw}]\bar{w}_{n+1}^2\bar{w}_n\\ +3[(NL)_{w7n}^{vdw}]\bar{w}_{n+1}\bar{w}_n^2 - [(NL)_{w8n}^{vdw}]\bar{w}_n^3\end{pmatrix}$$
$$+[(NL)_w^u]\{\bar{w}_n\bar{u}_n\} + [(NL)_w^v]\{\bar{w}_n\bar{v}_n\} + [(NL)_{w2}^w - \bar{F}_{e3}(NL_{2e})_w^w]\{\bar{w}_n^2\}$$
$$+[(NL)_{w3}^w - \bar{F}_{e4}(NL_{3e})_w^w]\{\bar{w}_n^3\} = \bar{F}_{we} + \bar{F}_{wn}$$
$$+\bar{F}_e\{((\bar{V}_{AC}cos\bar{\omega}\tau)^2 + 2\bar{V}_{AC}\bar{V}_{DC}cos\bar{\omega}\tau)(\bar{C}_4(NL_{3e})_w^w + \bar{C}_3(NL_{2e})_w^w + \bar{C}_2(K_e)_w^w + \bar{C}_1\bar{F}_1)\}$$

In Equation S26, for $n = 1$: $m_2 = q_1 = 0$; $q_2 = 1$; for $n = 2$: $m_2 = 0$; $m_1 = q_1 = 1$; $q_2 = -1$; and for $n = 3$: $q_2 = 0; m_1 = 1$; $q_1 = -1$. Also, $[(K)_w^{vdw}]_n$ is stiffness matrix for van der Walls effect. All coefficients of Equations S26–S28 are presented in Appendix 1 and 2.

Appendix 1

$$(M)_{un}^u = \iint \left(\chi_e\chi_i\vartheta_f\vartheta_j\right)d\xi\, d\theta\,, \qquad (K)_{un}^u = \iint \left(\alpha_{1n}\chi_e'\chi_i'\vartheta_f\vartheta_j + \alpha_{2n}\chi_e\chi_i\vartheta_f'\vartheta_j'\right)d\xi\, d\theta,$$
$$(K)_{un}^v = \frac{1}{2}\iint \left(\alpha_{3n}\chi_e'\phi_k\vartheta_f\alpha_l' + \alpha_{4n}\chi_e\phi_k'\vartheta_f'\alpha_l\right)d\xi\, d\theta\,,$$
$$(K)_{un}^w = \frac{1}{2}\iint \left(\alpha_{5n}\chi_e'\beta_o\vartheta_f\psi_l\right)d\xi\, d\theta,$$

$$(NL)_{un}^{w}=\frac{1}{2}\iint\left(\alpha_{6n}\chi_e'\beta_0'\beta_t'\vartheta_f\psi_p\psi_v+\alpha_{7n}\chi_e'\beta_o\beta_t\vartheta_f\psi_p'\psi_v'+\alpha_{8n}\chi_e\beta_0'\beta_t\vartheta_f'\psi_p\psi_v'\right)d\xi\,d\theta,$$

$$\bar{F}_{upn}=\frac{1}{2}\iint(\alpha_{26n}\chi_e'\vartheta_i)d\xi\,d\theta,\qquad (M)_{vn}^{v}=\iint\left(\phi_q\phi_k a_f a_l\right)d\xi\,d\theta$$

$$(K)_{vn}^{u}=\frac{1}{2}\iint\left(\alpha_{3n}\phi_q\chi_i'\alpha_f'\vartheta_l+\alpha_{4n}\phi_q'\chi_i\alpha_f\vartheta_l'\right)d\xi\,d\theta,$$

$$(K)_{vn}^{v}=\iint\left(\alpha_{9n}\phi_q\phi_k\alpha_f'\alpha_l'+\alpha_{13n}\phi_q'\phi_k'\alpha_f\alpha_l\right)d\xi\,d\theta,$$

$$(K)_{vn}^{w}=\frac{1}{2}\iint\left(\alpha_{12n}\phi_q\beta_o\alpha_f'\psi_l\right)d\xi\,d\theta,$$

$$(NL)_{vn}^{w}=\frac{1}{2}\iint\left(\alpha_{10n}\phi_q\beta_o\beta_t\alpha_g'\psi_p'\psi_v'+\alpha_{11n}\phi_q\beta_0'\beta_t'\alpha_g'\psi_p\psi_v+\alpha_{14n}\phi_q'\beta_0'\beta_t\alpha_g\psi_p\psi_v'\right)d\xi\,d\theta,$$

$$\bar{F}_{vpn}=\frac{1}{2}\iint\left(\alpha_{27n}\phi_q\alpha_f'\right)d\xi\,d\theta,$$

$$(M)_{wn}^{w}=\frac{1}{2}\iint\left(2\beta_r\beta_o\psi_s\psi_p+\alpha_{32n}\beta_r''\beta_o\psi_s\psi_p+\alpha_{33n}\beta_r\beta_o\psi_s''\psi_p+\bar{\rho}_{1n}\beta_r\beta_o\psi_s\psi_p\right)d\xi\,d\theta,$$

$$(C)_{wn}^{w}=\frac{1}{2}\iint\left(\bar{C}_{wn}\beta_r\beta_o\psi_s\psi_p+\bar{\rho}_{2n}\beta_r\beta_o'\psi_s\psi_p-\bar{\mu}_{1n}\beta_r\beta_o''\psi_s\psi_p-\bar{\mu}_{2n}\beta_r\beta_o\psi_s\psi_p''\right)d\xi\,d\theta,$$

$$(K)_{wn}^{u}=\frac{1}{2}\iint\left(\alpha_{5n}\beta_r\chi_i'\psi_s\vartheta_j\right)d\xi\,d\theta,$$

$$(K)_{wn}^{v}=\frac{1}{2}\iint\left(\alpha_{12n}\beta_r\phi_k\psi_s\alpha_l'\right)d\xi\,d\theta,$$

$$(K)_{wn}^{w}=\frac{1}{2}\iint\begin{pmatrix}2\alpha_{15n}\beta_r\beta_o\psi_s\psi_p+2\alpha_{21n}\beta_r''\beta_0''\psi_s\psi_p+2\alpha_{22n}\beta_o\beta_r\psi_s''\psi_p''\\+2\alpha_{23n}\beta_r'\beta_o'\psi_s'\psi_p'+\alpha_{24n}\beta_r\beta_o''\psi_s''\psi_p+\alpha_{24n}\beta_r''\beta_o\psi_s\psi_p''\\+2\alpha_{28n}\beta_r'\beta_o'\psi_s\psi_p+\bar{k}_w\beta_r\beta_o\psi_s\psi_p-\bar{k}_p\beta_r\beta_0''\psi_s\psi_p\\-\bar{k}_p m_0^2\beta_r\beta_o\psi_s\psi_p''-\bar{F}_{e2}(K_e)_w^w\\+\bar{\rho}_{3n}\beta_r\beta_0''\psi_s\psi_p-\bar{\mu}_{3n}\beta_r\beta_0'''\psi_s\psi_p-\bar{\mu}_{4n}\beta_r\beta_0'\psi_s\psi_p''\end{pmatrix}d\xi\,d\theta,$$

$$(K_e)_{wn}^{w}=\beta_r\beta_o\psi_s\psi_p,$$

$$(K)_{win}^{vdw}=\frac{1}{2}\iint\left(\beta_r\beta_o\psi_s\psi_p\right)d\xi\,d\theta,\qquad i=1\ldots4,$$

$$(NL)_{wn}^{u}=\frac{1}{2}\iint\begin{pmatrix}2\alpha_{6n}\beta_r'\beta_o'\chi_i'\psi_s\psi_p\vartheta_j+2\alpha_{7n}\beta_r\beta_o\chi_i'\psi_s'\psi_p'\vartheta_j\\+\alpha_{8n}\beta_r'\beta_o\chi_i\psi_s\psi_p'\vartheta_j'+\alpha_{8n}\beta_r\beta_o'\chi_i\psi_s'\psi_p\vartheta_j'\end{pmatrix}d\xi\,d\theta,$$

$$(NL)_{wn}^{v}=\frac{1}{2}\iint\begin{pmatrix}2\alpha_{10n}\beta_r\beta_o\phi_k\psi_s'\psi_p'\alpha_l'+2\alpha_{11n}\beta_r'\beta_o'\phi_k\psi_s\psi_p\alpha_l'\\+\alpha_{14n}\beta_r'\beta_o\phi_k'\psi_s\psi_p'\alpha_l+\alpha_{14n}\beta_r\beta_o'\phi_k'\psi_s'\psi_p\alpha_l\end{pmatrix}d\xi\,d\theta,$$

$$(NL)_{w2n}^{w}=\frac{1}{2}\iint\begin{pmatrix}\alpha_{19n}\beta_r\beta_o'\beta_t'\psi_s\psi_p\psi_v+2\alpha_{19n}\beta_r'\beta_o'\beta_t\psi_s\psi_p\psi_v+\alpha_{20n}\beta_r\beta_o\beta_t\psi_s\psi_p'\psi_v'\\+2\alpha_{20n}\beta_r\beta_o\beta_t\psi_s'\psi_p'\psi_v-\bar{F}_{e3}(NL_{2e})_w^w\end{pmatrix}d\xi\,d\theta\,,$$

$$(NL_{2e})_{wn}^{w}=\beta_r\beta_o\beta_t\psi_s\psi_p\psi_v,$$

$$(NL)_{w3n}^{w}=\frac{1}{2}\iint\left(\begin{array}{c}4\alpha_{16n}\beta_r'\beta_o'\beta_t'\beta_a'\psi_s\psi_p\psi_v\psi_b+4\alpha_{17n}\beta_r\beta_o\beta_t\beta_a\psi_s'\psi_p'\psi_v'\psi_b'\\+2\alpha_{18n}\beta_r'\beta_o'\beta_t\beta_a\psi_s\psi_p\psi_v'\psi_b'+2\alpha_{18n}\beta_r\beta_o\beta_t'\beta_a'\psi_s'\psi_p'\psi_v\psi_b\\-\bar{F}_{e4}(NL_{3e})_w^w\end{array}\right)d\xi\,d\theta$$

$$(NL_{3e})_{wn}^{w}=\beta_r\beta_o\beta_t\beta_a\psi_s\psi_p\psi_v\psi_b$$

$$(NL)_{win}^{vdw}=\frac{1}{2}\iint\left(\beta_r\beta_o\beta_t\beta_a\psi_s\psi_p\psi_v\psi_b\right)d\xi d\theta,\qquad i=1\ldots4,$$

$$\bar{F}_{wp}=\frac{1}{2}\iint(\alpha_{25}\beta_r\psi_s+\alpha_{30}\beta_r''\psi_s+\alpha_{31}\beta_r\psi_s'')d\xi\,d\theta,\qquad \bar{F}_1=\iint(\beta_r\psi_s)d\xi\,d\theta,$$

$$\bar{F}_{e1}=\frac{1}{2}\bar{F}_e\bar{F}_1,\ \ \bar{F}_{eDC}=\frac{1}{2}\bar{F}_e\bar{V}_{DC}^2,\bar{F}_{we}=\bar{C}_1\bar{F}_{eDC}\bar{F}_1,\ \ \bar{F}_{e2}=\bar{C}_2\bar{F}_{eDC},\bar{F}_{e3}=\bar{C}_3\bar{F}_{eDC},\bar{F}_{e4}=\bar{C}_4\bar{F}_{eDC},$$

Appendix 2

$$\alpha_{1n}=\frac{1}{m_{3n}}\bar{A}_{11n},\ \alpha_{2n}=\frac{m_{0n}^2}{m_{3n}}\bar{A}_{66n},\ \alpha_{3n}=\frac{m_{0n}}{m_{3n}}(\bar{A}_{12n}+\bar{A}_{21n}),\alpha_{4n}=\frac{2m_{0n}}{m_{3n}}\bar{A}_{66n},$$

$$\alpha_{5n}=\frac{m_{0n}}{m_{3n}}(\bar{A}_{12n}+\bar{A}_{21n}),\alpha_{6n}=\frac{1}{m_{1n}m_{3n}}\left(\bar{A}_{11n}-\bar{\tau}_{0n}^{ps}-\bar{\tau}_{0n}^{NI}\right),\ \alpha_{7n}=\frac{m_{0n}m_{2n}}{2m_{3n}}(\bar{A}_{12n}+\bar{A}_{21n}),$$

$$\alpha_{8n}=\frac{2m_{0n}m_{2n}}{m_{3n}}\bar{A}_{66n},\ \alpha_{9n}=\frac{m_{0n}^2}{m_{3n}}\bar{A}_{22n},\ \alpha_{10n}=\frac{m_{0n}^2m_{2n}}{m_{3n}}\left(\bar{A}_{22n}-\bar{\tau}_{0n}^{ps}-\bar{\tau}_{0n}^{NI}\right),$$

$$\alpha_{11n}=\frac{m_{2n}}{2m_{3n}}(\bar{A}_{12n}+\bar{A}_{21n}),\ \alpha_{12n}=\frac{2m_{0n}^2}{m_{3n}}\left(\bar{A}_{22n}-\bar{\tau}_{0n}^{ps}-\bar{\tau}_{0n}^{NI}\right),\alpha_{13n}=\frac{1}{m_{3n}}\bar{A}_{66n},$$

$$\alpha_{14n}=\frac{2m_{2n}}{m_{3n}}\bar{A}_{66n},\ \alpha_{15n}=\frac{m_{0n}^2}{m_{3n}}\left(\bar{A}_{22n}-2\left(\bar{\tau}_{0n}^{ps}+\bar{\tau}_{0n}^{NI}\right)\right),\ \alpha_{16n}=\frac{1}{4m_{1n}^2m_{3n}}\left(\bar{A}_{11n}-2\left(\bar{\tau}_{0n}^{ps}+\bar{\tau}_{0n}^{NI}\right)\right),$$

$$\alpha_{17n}=\frac{m_{0n}^2m_{2n}^2}{4m_{3n}}\left(\bar{A}_{22n}-2\left(\bar{\tau}_{0n}^{ps}+\bar{\tau}_{0n}^{NI}\right)\right),\alpha_{18n}=\frac{m_{2n}^2}{4m_{3n}}(4\bar{A}_{66n}+\bar{A}_{12n}+\bar{A}_{21n}),$$

$$\alpha_{19n}=\frac{m_{2n}}{2m_{3n}}(\bar{A}_{12n}+\bar{A}_{21n}),\alpha_{20n}=\frac{m_{0n}^2m_{2n}}{m_{3n}}\left(\bar{A}_{22n}-2\left(\bar{\tau}_{0n}^{ps}+\bar{\tau}_{0n}^{NI}\right)\right),$$

$$\alpha_{21n}=\frac{1}{m_{1n}^2m_{3n}}(\bar{D}_{11n}-\bar{E}_{11n}),\ \alpha_{22n}=\frac{m_{0n}^2m_{2n}^2}{m_{3n}}(\bar{D}_{22n}-\bar{E}_{11n}),\ \alpha_{23n}=\frac{4m_{2n}^2}{m_{3n}}\bar{D}_{66n},$$

$$\alpha_{24n}=\frac{m_{2n}^2}{m_{3n}}(\bar{D}_{12n}+\bar{D}_{21n}-2\bar{E}_{11n}),\alpha_{25n}=\frac{m_{0n}m_{1n}}{m_{3n}}\left(2\left(\bar{\tau}_{0n}^{ps}+\bar{\tau}_{0n}^{NI}\right)-\bar{N}_{\theta pn}\right),$$

$$\alpha_{26n}=\frac{m_{1n}}{m_{3n}}\left(2\left(\bar{\tau}_{0n}^{ps}+\bar{\tau}_{0n}^{NI}\right)-\bar{N}_{xpn}\right),\alpha_{27n}=\frac{m_{0n}m_{1n}}{m_{3n}}\left(2\left(\bar{\tau}_{0n}^{ps}+\bar{\tau}_{0n}^{NI}\right)-\bar{N}_{\theta pn}\right),$$

$$\alpha_{28n}=\frac{1}{2m_{3n}}\left(2\left(\bar{\tau}_{0n}^{ps}+\bar{\tau}_{0n}^{NI}\right)-\bar{N}_{xpn}\right),\alpha_{29n}=\frac{m_{0n}^2}{2m_{3n}}\left(2\left(\bar{\tau}_{0n}^{ps}+\bar{\tau}_{0n}^{NI}\right)-\bar{N}_{\theta pn}\right),$$

$$\alpha_{30n}=\frac{1}{m_{3n}}\bar{M}_{xpn},\alpha_{31n}=\frac{m_{0n}^2}{m_{3n}}\bar{M}_{\theta pn},\alpha_{32n}=\frac{1}{2m_{1n}^2m_{3n}}\bar{G}_{11n}^*,\alpha_{33n}=\frac{m_{2n}^2}{2m_{3n}}\bar{G}_{11n}^*,$$

References

[1] Gurtin, M. E.; Murdoch, A. I. *Int. J. Solids Struct.* **1978,** *14,* 431–440. doi:10.1016/0020-7683(78)90008-2

[2] Hashemi Kachapi, S. H.; Dardel, M.; Mohamadi Daniali, H.; Fathi, A. *Proc. Inst. Mech. Eng., Part C* **2019,** *233,* 5264–5279. doi:10.1177/0954406219845019

[3] Hashemi Kachapi, S. H.; Dardel, M.; Mohamadi Daniali, H.; Fathi, A. *Thin-Walled Structures* **2019,** *143,* 106210. doi:10.1016/j.tws.2019.106210

[4] Hashemi Kachapi, S. H.; Dardel, M.; Mohamadi Daniali, H.; Fathi, A. *Appl. Math. Modell.* **2019,** *75,* 279–309. doi:10.1016/j.apm.2019.05.035

[5] Hashemi Kachapi, S. H.; Dardel, M.; Mohamadi Daniali, H.; Fathi, A. *J. Vib. Control* **2019,** *26,* 680–700. doi:10.1177/1077546319889858

[6] Jafari, S. B.; Malekfar, R.; Khadem, S. E. *Int. J. Nanosci. Nanotechnol.* **2011,** *7,* 137–142.

[7] He, X. Q.; Kitipornchai, S.; Liew, K. M. *J. Mech. Phys. Solids* **2005,** *53,* 303–326. doi:10.1016/j.jmps.2004.08.003

[8] Wang, L.; Ni, Q. A. *Mech. Res. Commun.* **2009,** *36,* 833–837. doi:10.1016/j.mechrescom.2009.05.003
